# Using signals associated with safety in avoidance learning: computational model of sex differences

**DOI:** 10.7717/peerj.1081

**Published:** 2015-07-14

**Authors:** Milen L. Radell, Kevin D. Beck, Kevin C.H. Pang, Catherine E. Myers

**Affiliations:** 1 Neurobehavioral Research Laboratory, VA New Jersey Health Care System, East Orange, NJ, USA; 2 Department of Pharmacology, Physiology & Neuroscience, New Jersey Medical School, Rutgers University, Newark, NJ, USA

**Keywords:** Avoidance, Safety signal, Anxiety vulnerability, Associative learning, Anxiety disorders, Sex differences, Computational model

## Abstract

Avoidance behavior involves learning responses that prevent upcoming aversive events; these responses typically extinguish when the aversive events stop materializing. Stimuli that signal safety from aversive events can paradoxically inhibit extinction of avoidance behavior. In animals, males and females process safety signals differently. These differences help explain why women are more likely to be diagnosed with an anxiety disorder and exhibit differences in symptom presentation and course compared to men. In the current study, we extend an existing model of strain differences in avoidance behavior to simulate sex differences in rats. The model successfully replicates data showing that the omission of a signal associated with a period of safety can facilitate extinction in females, but not males, and makes novel predictions that this effect should depend on the duration of the period, the duration of the signal itself, and its occurrence within that period. Non-reinforced responses during the safe period were also found to be important in the expression of these patterns. The model also allowed us to explore underlying mechanisms for the observed sex effects, such as whether safety signals serve as occasion setters for aversive events, to determine why removing them can facilitate extinction of avoidance. The simulation results argue against this account, and instead suggest the signal may serve as a conditioned reinforcer of avoidance behavior.

Avoidance behavior involves learning to perform a response to cause the omission of an expected aversive event. In the typical paradigm used to study avoidance learning, the subject is presented with a stimulus (W) that serves as a warning for a subsequent aversive event (U, e.g., electric shock). Over trials, rats can learn to perform a response (e.g., pressing a lever) to avoid U. Strong avoidance learning that is resistant to extinction is a prominent feature of most anxiety disorders and post-traumatic stress disorder (PTSD) (Rauch, Shin & Phelps, 2006; American Psychiatric Association, 2013), therefore animal models with extinction resistance and computational modeling can be used to understand the neural underpinning of these conditions. Treatment for anxiety often involves an exposure-based approach that is analogous to extinction training where W is no longer followed by U (Bouton, 1988; Lovibond et al., 2009). That is, the patient is exposed to stimuli or situations previously associated with threat until they no longer elicit fear. However, behaviors that lead to perceived safety, such as looking away from a threatening stimulus (Wells et al., 1995), can interfere with learning that no aversive consequence will follow. Instead, the absence of an aversive consequence may be attributed to performing the avoidance behavior, reducing treatment effectiveness (McManus, Sacadura & Clark, 2008; Salkovskis et al., 1999; Wells et al., 1995). For instance, Lovibond et al. (2009) trained participants to press a button in order to avoid electric shock. Later, when shock was no longer delivered, those who were prevented from performing the avoidance response extinguished faster as shown by reduced skin conductance responses and lower shock expectancy ratings (Lovibond et al., 2009).

Similar to avoidance responses, cues associated with the absence of threat (i.e., potential safety signals) can also confer protection from extinction, which has been observed in human fear conditioning (Lovibond, Davis & O’Flaherty, 2000) and in both human (Sheynin et al., 2014) and animal (Beck et al., 2011) avoidance learning. Beck et al. (2011) found that the omission of a stimulus previously associated with a safe inter-trial interval (ITI), enhanced the extinction of avoidance behavior in female, but not male, Sprague Dawley rats relative to animals that were never exposed to this ITI signal (Beck et al., 2011; Catuzzi & Beck, 2014). On the other hand, the ITI signal did not alter the rate of acquisition in either sex (Beck et al., 2011). Although some rat strains may be more susceptible to using such a signal (Beck et al., 2011; Catuzzi & Beck, 2014), it was only the omission of the ITI signal during extinction that was important, causing female rats to extinguish faster at a rate similar to that of male rats. One recent study found that ITI signals can also affect avoidance in humans, and that males and females are differentially impacted (Sheynin et al., 2014). These results suggest that sex differences in using signals associated with safe periods could confer vulnerability to anxiety disorders by rendering avoidance behavior more difficult to extinguish in females. This is important considering that women are more likely to be diagnosed with an anxiety disorder, and PTSD, and exhibit differences in symptom presentation and course, compared to men (Altemus, Sarvaiya & Epperson, 2014). Therefore, understanding sex differences in the conditions under which stimuli can come to protect against extinction could provide the opportunity to tailor treatment to the individual and improve clinical outcomes.

The purpose of the current study was to simulate avoidance learning in outbred male and female Sprague Dawley (SD) rats to understand how the sex differences in ITI signal use reported by Beck et al. (2011) arise. While avoidance learning in rodents has been shown to depend on individual differences, including strain (Beck et al., 2010; Berger & Starzec, 1988; Bond, 1981; Kuribara, 1982; Servatius et al., 2008; Sutterer, Perry & DeVito, 1980) and sex (Beatty & Beatty, 1970; Beck et al., 2010; Dalla & Shors, 2009; Heinsbroek et al., 1983; Scouten, Groteleuschen & Beatty, 1975; Van Oyen, Walg & Van De Poll, 1981), these studies have not examined sex differences in ITI signal use. A number of studies have also examined the role of ITI signals through a diverse set of paradigms, including shuttle box, running wheel, running alley, lever press and jumping avoidance (Bolles, Grossen & Bond, 1969; Cándido et al., 1991; Katzev & Hendersen, 1971). However, differences in the training procedure between studies along with the type and nature of the ITI signals used make it difficult to determine under what conditions such signals modulate avoidance learning. Additionally, most of these studies focus on acquisition. The study by Beck et al. (2011) was chosen to be the focus of the current modeling effort because it presents data on the acquisition and extinction of avoidance from both males and females using the same paradigm.

A computational approach was used to (1) test several hypotheses about why omission of the ITI signal can enhance the extinction of avoidance behavior in female, but not male, SD rats, (2) understand the underlying mechanisms that might differentiate ITI signal processing in males and females, and (3) generate predictions that can be examined by future experiments. The model was able to account for sex differences in avoidance learning via changes in only two parameters, each associated with different components of the behavior. The results of the simulations also make several novel predictions, specifically that whether omission of the ITI signal will facilitate extinction in females, but not males, depends on the duration of the ITI, the duration of the ITI signal itself, and its timing within the interval, and suggest the signal can serve as a positive or negative reinforcer of avoidance behavior under different conditions.

## General Method

**Figure 1:**
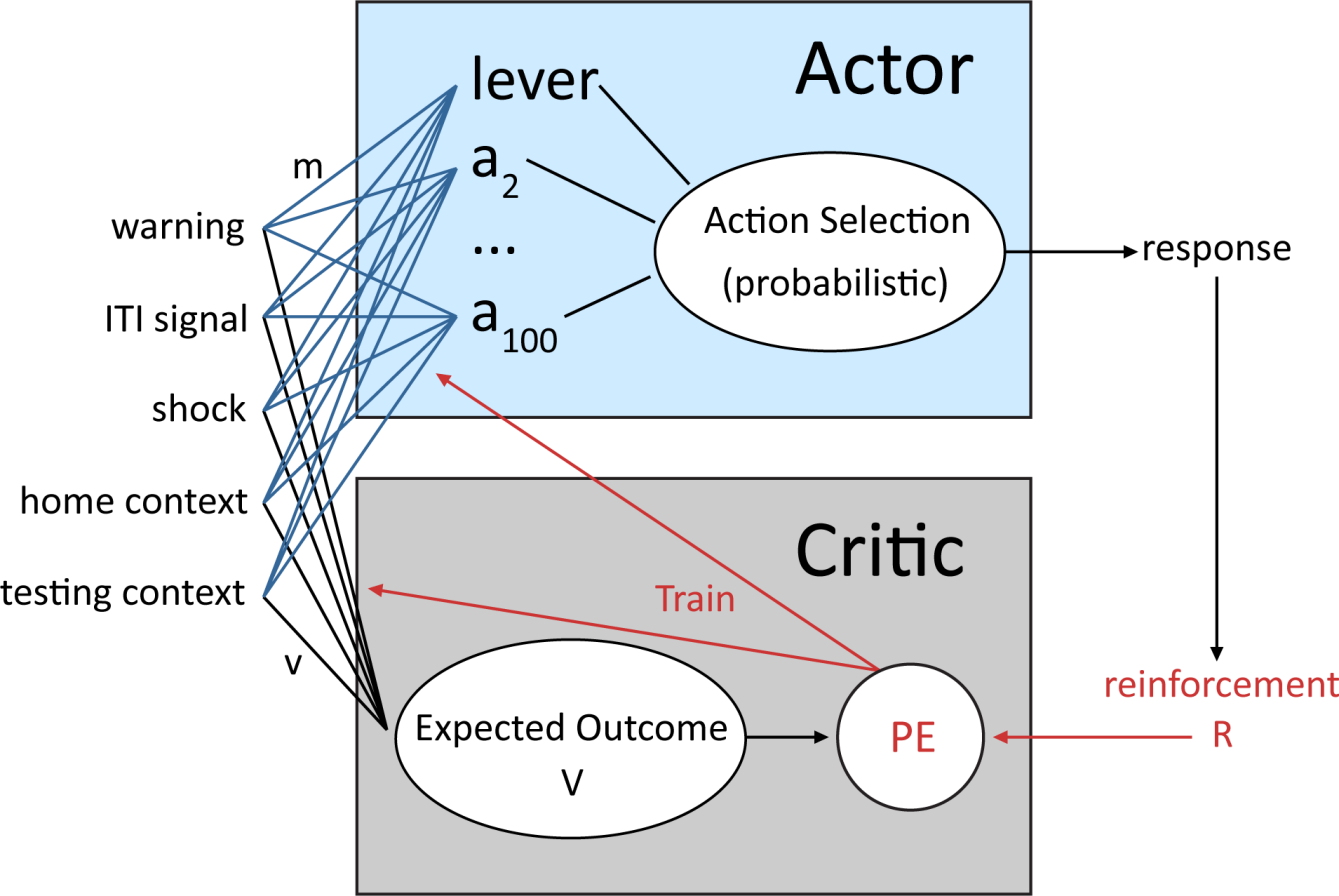
Schematic of the reinforcement learning model used in the current study adapted from Myers et al. (2014). An actor and critic module receive inputs representing external stimuli, which make up the current state. The critic evaluates each possible state based on the value of past, current and predicted future outcomes (reinforcement value, *R*) of that state to compute a prediction error (PE) signal that is then used to adjust the probability of each response that the actor module can select. Through trial-and-error, actions that minimize aversive events (i.e., lever press) become more probable.

A reinforcement learning model with probabilistic action selection previously shown to account for active avoidance behavior (Myers et al., 2014), was used to simulate the lever press avoidance paradigm employed by Beck et al. (2011). The model belongs to a class of temporal difference reinforcement learning models that consist of an actor and critic module (Montague, Hyman & Cohen, 2004; Barto et al., 1983). The architecture of the model, based on that of Myers et al. (2014), is shown in Fig. 1. The critic receives inputs that represent a configuration of internal and external stimuli, together defined as the current state. Each of the possible states is followed by an outcome (i.e., reward or punishment) that the critic uses to evaluate that state, computing its reward value (Myers et al., 2014). This value is then used to adjust the probability of each response that the actor module can make when the same configuration of inputs is presented in the future. Through trial and error, actions (i.e., lever press) that minimize aversive events become more common, while other possible actions become less probable (Dayan & Balleine, 2002). In addition to the immediate outcome of each action, response selection is also influenced by the prior history of reinforcement and the predicted future value of that action (Barto, 1995; Dayan & Balleine, 2002; Sutton, 1988). Thus, the learning mechanism in the model is based on several theories of avoidance learning, including two-factor theory (Maia, 2010; Mowrer, 1951), where avoidance is due to both stimulus–stimulus and stimulus-outcome associations, and cognitive expectancy theories, where action selection is based on expectations about future outcomes (Seligman & Johnston, 1973; Tolman, 1932).

This type of model was chosen because it captures key elements common to many past and current models of reinforcement learning (Barto, 1995). In addition, the actor and critic resemble some features of the anatomy and physiology of the basal ganglia thought to be important for both appetitive and aversive learning. For instance, the temporal difference prediction error signal in the critic module maps onto dopamine neuron activity (for review, see Joel, Niv & Ruppin, 2002). It can reproduce a number of temporal features of phasic dopamine neuron firing including increases in activity when reward is expected, but decreases below baseline levels when an expected reward does not occur (Joel, Niv & Ruppin, 2002; Suri, 2002). While dopaminergic neurotransmission is typically associated with appetitive learning, a subset of dopamine neurons may also play a role in aversive learning (Schultz, 2010; Matsumoto & Hikosaka, 2009). The prediction error signal could also be related to serotonergic inputs to the striatum (Moutoussis et al., 2008). Similar models have been widely used by many researchers to understand the roles of brain substrates, such as the nigrostriatal dopamine system, the dorsal striatal action selection system, the prefrontal cortex, and the hippocampus (Moustafa, Myers & Gluck, 2009; Moustafa et al., 2010) and a number of psychiatric and neurological disorders (Maia & Frank, 2011). More recently, these models have also been used to understand avoidance learning and avoidance vulnerability in both animals (Myers et al., 2014) and humans (Sheynin et al., 2015).

In particular, our prior simulations using this model showed it could explain several key differences in avoidance learning between the SD and avoidance-susceptible Wistar Kyoto (WKY) rodent strains, including faster acquisition, resistance to extinction and the lack of warm-up between training sessions in the WKY rats (Myers et al., 2014). In particular, the lack of warm-up, which is a phenomenon where avoidance responding is lower at the start of a session relative to the end of the previous session, could confer resistance to extinction in the WKY strain because greater avoidance responding precludes learning that shock is no longer present during extinction (Servatius et al., 2008). The model suggested that the absence of warm-up in the WKY strain was due to changes in the tendency to explore new behaviors vs. perform those that were reinforced in the past, the tendency to repeat previous behaviors regardless of reinforcement history, and how much prior experiences change predictions about future outcomes (Myers et al., 2014). The model also made several predictions for future research, one of which was subsequently tested and confirmed empirically (Myers et al., 2014). Actor-critic temporal difference models can, therefore, contribute to understanding avoidance learning, including changes in avoidance behavior associated with PTSD (faster acquisition and resistance to extinction), and generate testable predictions for future studies in both animals and humans.

### Simulation design

The lever press avoidance protocol used by Beck et al. (2011) consisted of an acquisition and extinction phase. The acquisition phase was divided into 240 trials, each including a 60-s warning period marked by a tone, that was followed by scrambled 0.5-s shocks at 1 mA delivered through the floor every 3.5 s for a maximum of 99 shocks. The warning stimulus remained on throughout the shock period. On a given trial, rats learned to press a lever to escape the shock once it was on, or avoid it altogether by pressing the lever during the warning period just prior to shock. Upon successful escape or avoidance, or after all 99 shocks were delivered, both the warning stimulus and the shock (if present) were terminated. This was followed by a safe inter-trial interval (ITI) that was, for half of the subjects, marked by the onset of a flashing light serving as an ITI signal. The extinction phase was identical to acquisition, except that the shock and ITI signal were never presented. A daily training session consisted of twenty trials, and began with a 60-s stimulus-free period in the testing chamber. Sessions took place every other day, 48 h apart, with animals removed to the home cage between sessions.

**Figure 2:**
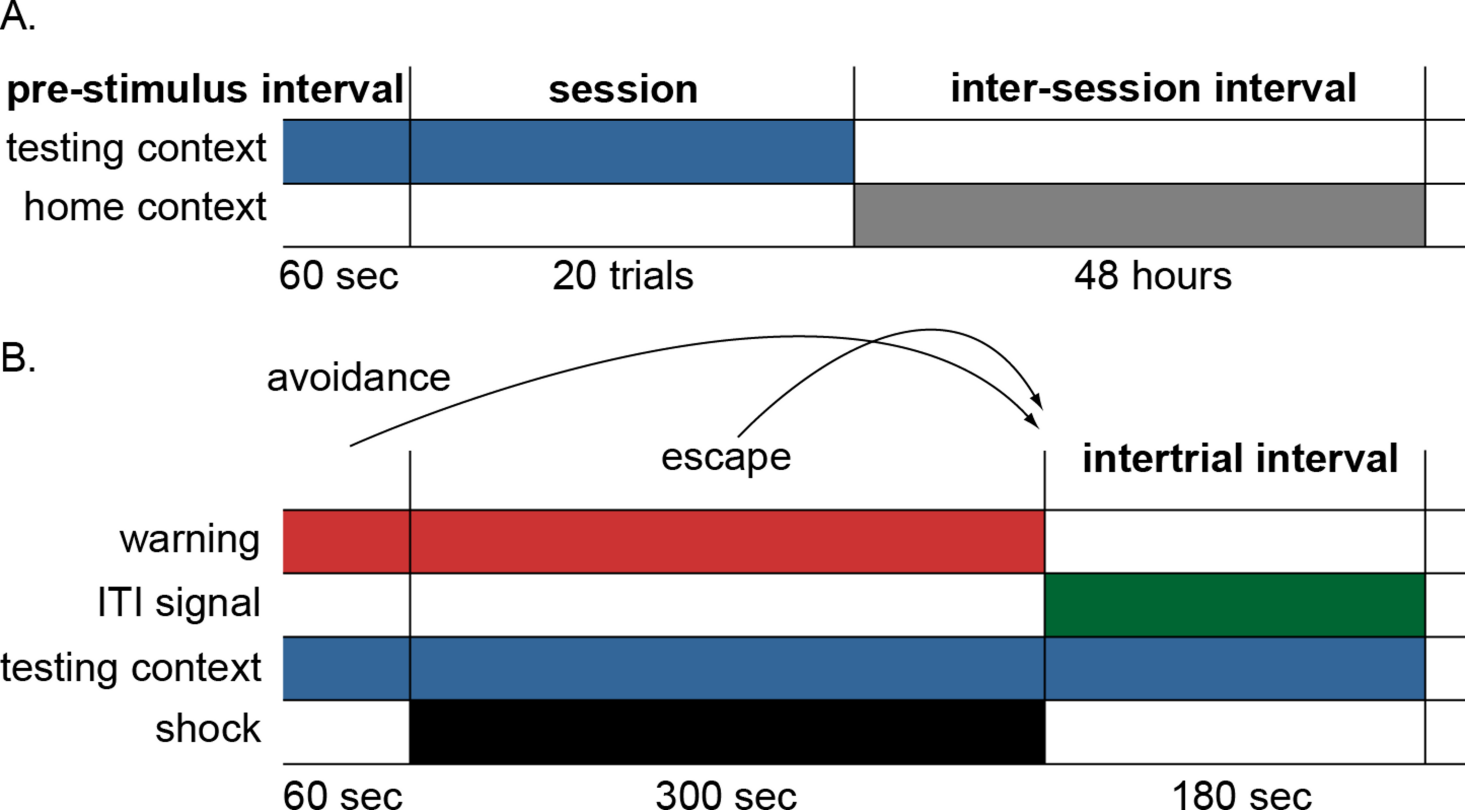
Schematic of the simulation design. (A) Each simulation run consisted of 12 sessions divided into 20 trials with a 48-hour interval between sessions. Each session was preceded by a 60-sec pre-stimulus interval where only the testing context input was presented simulating time animals spent habituating to the testing cage. The end of the session was marked by the offset of the testing context and the onset of the home context input, representing time spent in the home cage between sessions. (B) Schematic of events within each trial. On each trial, the warning signal appeared for 60 s and remained on for 300 s during which the shock occurred. Lever press responses made during the warning period (prior to presentation of shock) were scored as avoidance responses, terminated the warning signal, and caused immediate initiation of the intertrial interval. Lever press responses made during the shock period were scored as escape responses and terminated both warning signal and shock, causing immediate initiation of the intertrial interval. Finally, those made during the intertrial interval were scored as intertrial responses. In some conditions, an ITI signal was present during the intertrial interval; the testing context was present throughout the testing session. The actor module selected a response among multiple possible actions, including lever press, every timestep (1 timestep = 10 s).

The procedure used to simulate this paradigm was similar to that of Myers et al. (2014). Stimuli were represented as binary inputs to both the actor and critic modules indicating if each stimulus was present (1) or absent (0) on a given timestep. The stimuli included warning signal, shock, ITI signal, testing context and home context. Context inputs represented the testing and home cage of an animal, respectively. There were 12 sessions in the acquisition phase and each began with a 6-timestep pre-stimulus period where only the testing context input was present (Fig. 2A). Each session was further divided into 20 trials. Every trial was divided into 54 timesteps, each representing 10 s of simulated time (Fig. 2B). The start of every trial was marked by the onset of the warning input, which continued for 6 timesteps. At that point the shock input also became active and could continue (along with the warning) for up to 30 timesteps. At each timestep, the actor could select a response from a set of possible actions that included lever press. Press responses during the warning period were scored as avoidance, while those during the shock interval were scored as escape. A lever press resulted in the immediate termination of both the warning signal and shock (if present) and transition to an 18-timestep ITI that, for some of the simulations, could be marked by the onset of the ITI signal. As a secondary measure of avoidance behavior, the latency to lever press was also recorded for each trial. If no press response occurred, the latency was scored as the maximum number of timesteps in the trial (i.e., 54). Finally, at the end of each session, an 18,000-timestep inter-session interval was simulated corresponding to the 48 h animals spent in the home cage prior to the next session. During this time, the only input presented to the model was the home context. All simulation figures report the mean of 10 simulations per group, and error bars represent standard error around the mean.

### Actor module

At each timestep *t*, the actor selected a response *r* from a set of 100 possible actions. One of these actions was designated as the lever press while the remaining actions represented arbitrary behaviors, such as rearing or grooming, that an animal may engage in besides pressing the lever. The probability of selecting a response *r* at timestep *t* was defined as

\begin{eqnarray*} P r(r)=\frac{f\left(\frac{{M}_{r}}{T}\right)}{\sum _{a}f\left(\frac{{M}_{a}}{T}\right)} \end{eqnarray*}

where *f*(*x*) = *e^x^* for *a* = 1–100. The explore/exploit parameter *T* determined the tendency to repeat actions that were previously reinforced vs. explore the effect of new actions, and was set to 1.0 for all simulations. This value was chosen because it was found to best simulate SD rat behavior in prior modeling work (Myers et al., 2014). In contrast, lower values increase the tendency to repeat previously selected responses—a behavioral pattern associated with high behavioral inhibition and typical in the WKY rat strain (Myers et al., 2014). At each timestep *t*, the values *M_a_* were defined as

\begin{eqnarray*} {M}_{a}=\sum _{i}{m}_{a i}{I}_{i}+{c}_{a i}P \end{eqnarray*}

where *I_i_* was the current value of input *i* and *m_ai_* was the strength of connection from input *i* to action *a* and *c* was a working memory trace that recorded prior actions in response to a particular configuration of inputs: *c_ri_* = 1 for the action *r* executed at time *t* while *c_ai_* = 0.95*c_ai_* for all other actions *a* ≠ *r*. All values of *m* had an initial value of 0.01 while those of *c* started at 0 at the beginning of a simulation. Finally, the perseveration parameter *P* controlled the tendency to repeat a prior response and was set to 0.25 for all simulations.

### Critic module

External reinforcement *R* was provided depending on the action *r* selected by the actor at timestep *t*. If there was shock at time *t* + 1 then *R* was set to *R*_shock_, a large negative value (i.e., −4 or −8 depending on sex as described in the next section). Otherwise, *R* was set to 0 unless the selected action was lever press, in which case it was set to −0.2, a small negative value representing the energy cost associated with that response. Based on *R*, the critic module computed prediction error *PE* via

\begin{eqnarray*} P E=R+\gamma V-{V}^{{\prime}} \end{eqnarray*}

where *γ* was the discounting factor, *V* was the predicted future value of *R*, calculated as

\begin{eqnarray*} V=\sum _{i}{v}_{i}{I}_{i} \end{eqnarray*}

and where *V*′ was the value of *V* from the previous time *t*. The value of *γ* was held constant at 0.9 as in previous simulations of avoidance learning in SD rats using the same model (Myers et al., 2014). Selection of this value was motivated by prior research, which has estimated it to be closer to 1 based on how much dopamine neuron responses decrease with longer stimulus-reinforcement intervals (Suri & Schultz, 1999; Suri, 2002). Additionally, values closer to 1 allow more distant past reinforcement to influence future decisions, leading to learning that is based on both short- and long-term consequences of actions (Barto, 1995). In contrast, a value closer to 0 results in learning only based on short-term consequences, which carries several disadvantages, including reliance on immediate primary reinforcement, which may not be always available, and can preclude selecting actions that lead to greater future reinforcement (Barto, 1995). Finally, in calculating *V*, all values of *v_i_* were set to 0 at the start of a simulation and were updated as

\begin{eqnarray*} \Delta {v}_{i}=\hspace{0.167em} \alpha \times P E\times {I}_{i} \end{eqnarray*}

where *α* was a learning rate that determined the rate of weight change in the critic, and was set to 0.01 or 0.05 depending on sex as described in the next section. Our prior simulations using this model showed that changes in *α* can affect the rate of extinction, largely independent of acquisition (Myers et al., 2014). The values of *v_i_* could not exceed ±*R*_shock_ to prevent them from growing out of bounds.

The weights in the actor module *m_ri_* for the chosen action *r* were also updated based on *PE* via

\begin{eqnarray*} \Delta {m}_{r i}=\varepsilon \times (P E-{m}_{r i})\times {I}_{i} \end{eqnarray*}

where ε was a learning rate that determined the rate of weight change in the actor, and was held constant at 0.005 as in previous simulations of avoidance learning in SD rats using the same model (Myers et al., 2014). The values of *m* could only be ≥0.

### Simulating sex differences

The parameter values chosen for male simulations in the current study were identical to those in our previous simulations of male SD behavior (Myers et al., 2014). To simulate female sex, we varied the value of two parameters in the model—shock cost and the learning rate in the critic (*α*).

Shock cost was set to −8 to simulate females, compared to −4 to simulate males, where greater negative values represent more aversive shock. This manipulation is analogous to changes in shock intensity and was chosen because female rats appear to be more sensitive to shock than males (Pare, 1969) and these differences have also been associated with faster avoidance learning in females (Beatty & Beatty, 1970). Faster acquisition of avoidance behavior in females was also reported by Beck et al. (2011) and greater shock cost has been shown to speed up acquisition in prior simulations using this model (Myers et al., 2014).

In addition to shock cost, the learning rate in the critic module was set to 0.01 for female simulations, compared to 0.05 for males. This manipulation was chosen because male SD rats have greater D1 dopamine receptor density in the nucleus accumbens than female SD rats (Andersen et al., 1997), which could correspond to a higher learning rate in the model. While there are a number of sex differences in the release, uptake or sensitivity to dopamine in the ventral striatum (Becker, Perry & Westenbroek, 2012), we chose to focus on one to maintain simplicity, and because this difference also appears to have a direct correlate in the model. As discussed earlier, the prediction error signal computed by the critic module reproduces a number of temporal features of phasic dopamine neuron activity (Suri, 2002), and could correspond to dopaminergic inputs to the striatum (Collins & Frank, 2014; Moutoussis et al., 2008; Seymour et al., 2004). Also, since dopaminergic signals may serve a gating function (Montague, Hyman & Cohen, 2004), when the learning rate is higher, this could “open the gate”, allowing the current goal representation (or policy of the model) to be updated. In contrast, lower learning rates may preserve the current policy in the face of interference, consistent with the resistance to extinction observed in female SD rats (Beck et al., 2011).

## Experiment 1: Sex Differences in ITI Signal Use

**Figure 3:**
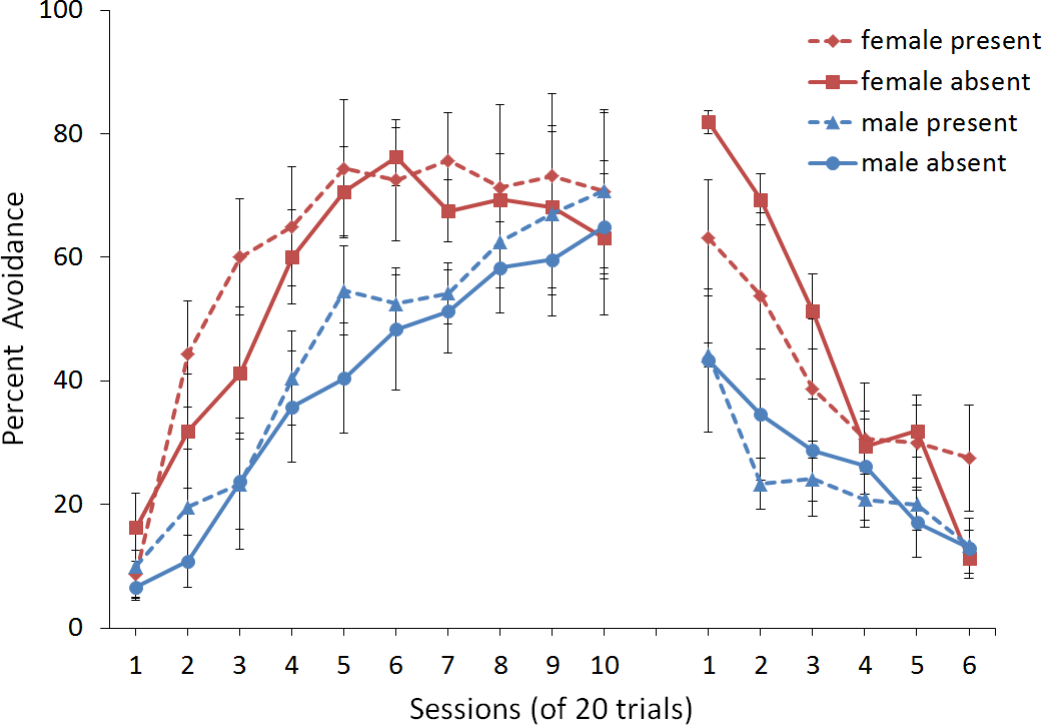
Mean percent avoidance responses for male and female Sprague Dawley rats in active lever-press avoidance. An ITI signal was either present or absent during acquisition but was never presented during extinction. Females learned to avoid significantly faster than males. Avoidance in either sex was not affected by the ITI signal during acquisition. The subsequent omission of the ITI signal led to faster extinction in female rats trained with this signal during acquisition relative to female rats that were never exposed to the signal. In contrast, omission of the signal had no effect on extinction rate in male rats. Error bars represent SEM. Image adapted from Beck et al. (2011); also see Catuzzi & Beck (2014).

First, we attempted to replicate the sex differences in ITI signal use observed in SD rats (Beck et al., 2011). That study considered male and female SD rats, assigned to one of two conditions based on the presence of an ITI signal. Thus, for one condition the ITI signal was present during acquisition but not extinction (signal present), while for the other condition, there was no ITI signal (signal absent). Results showed that female rats acquired the avoidance response faster than male rats, regardless of ITI signal condition, while female rats (but not male rats) extinguished faster in the signal present than in the signal absent condition (Fig. 3).

One interpretation of these results is that adding or removing the signal could provide general information that the task has changed, enhancing discriminability between states of danger and safety, which should facilitate learning not to respond during extinction (Mineka & Oehlberg, 2008). If so, presenting the ITI signal for the first time during extinction should be similar to presenting it during acquisition then omitting it during extinction. In either case, the change would indicate that the task has changed, potentially reducing the likelihood of lever press responses. This prediction, which has not been examined in rats, was also tested in the model.

### Modeling methods

One set of simulations were trained following the avoidance protocol described in the general methods; half were “male” and half were “female,” based on shock cost and learning rates as defined earlier. Groups were further divided by ITI condition, where an ITI signal was either present or absent in acquisition. In either case, the signal was never present in extinction. A second set of simulations were trained following the same procedure as above; however, the ITI signal was now always present in extinction, whether or not it had occurred during acquisition. If the ITI signal only provides general information as an unexpected event, extinction should be faster when the ITI changes (added or deleted) from acquisition to extinction, compared to conditions where the ITI is always present or absent in both phases.

### Results and discussion

**Figure 4:**
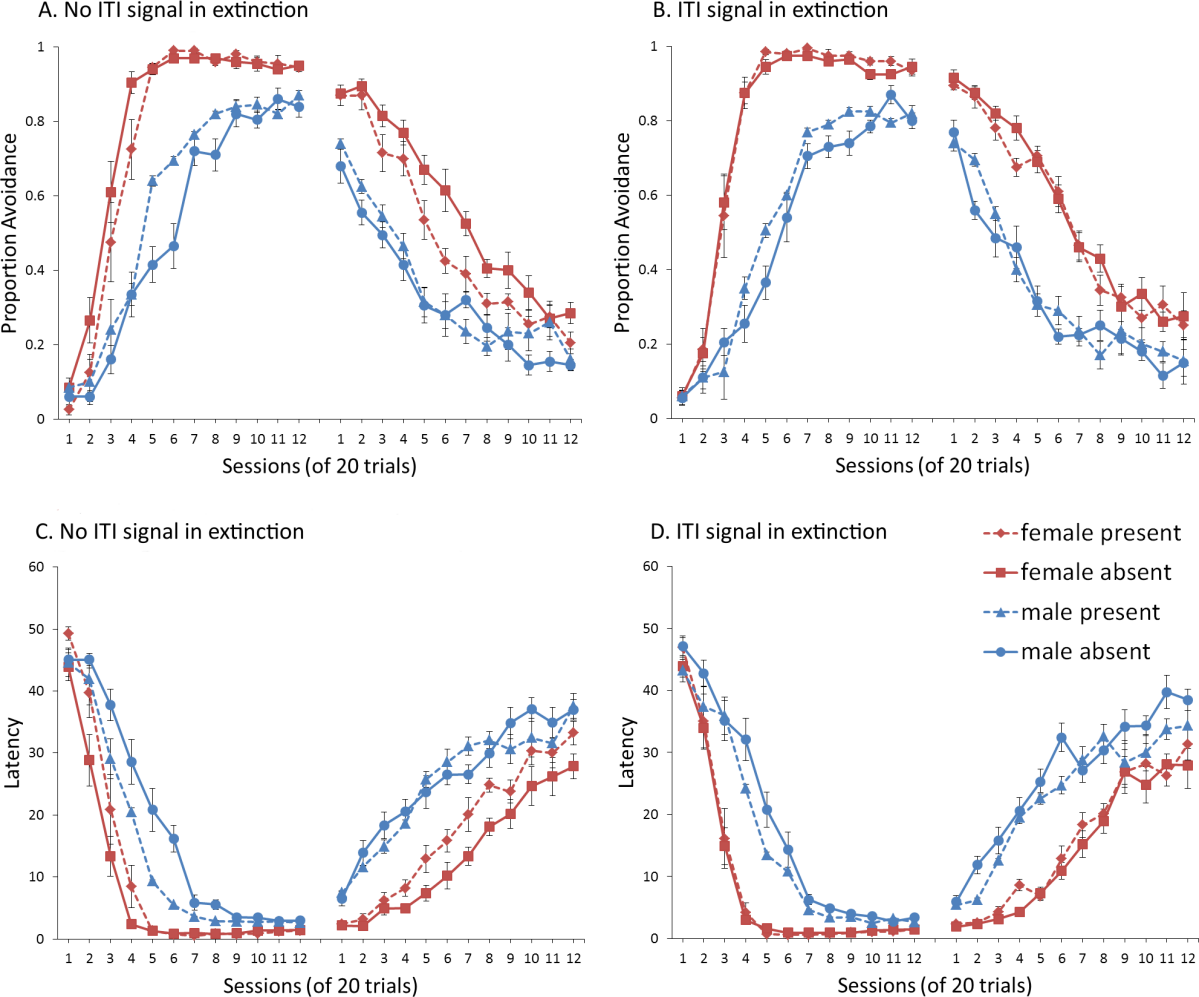
Mean proportion avoidance responses and latency to press the lever for simulations (*n* = 10 per group) in Experiment 1. Sex differences were simulated via changes in two parameters—shock cost (set to −4 for male and −8 for female simulations) and the learning rate in the critic (set to 0.05 for male and 0.01 for female simulations). An ITI signal was present or absent in acquisition, and either always absent (A and C) or always present (B and D) in extinction. Errors bars represent SEM.

A mixed-design ANOVA with within-subjects factor of session, and between-subjects factors of sex (male or female) and ITI signal condition (present or absent), was performed on the proportion of avoidance responses. The acquisition and extinction phases were analyzed separately. Separate analyses were also performed on simulations in which an ITI signal was either never presented (Fig. 4A), or always presented (Fig. 4B), in the extinction phase. For a complete summary of statistics for these simulations, refer to the Supplemental Information 2, Section 1. Here, and in all subsequent experiments, only the most relevant significant differences will be discussed in the main text.

Simulations in which an ITI signal was never presented during extinction (Fig. 4A) replicate three key aspects of the empirical data on SD avoidance (see Beck et al., 2011; Catuzzi & Beck, 2014). First, female simulations acquired the avoidance response faster than male simulations, and reached higher overall performance, regardless of condition. This was confirmed by a significant interaction between session and sex, *F*(3.91, 140.6) = 19.589, *p* < .001, ${\eta }_{p}^{2}=.352$. There were also main effects of session, *F*(3.91, 140.6) = 310.105, *p* < .001, ${\eta }_{p}^{2}=.896$, and sex, *F*(1, 36) = 161.711, *p* < .001, ${\eta }_{p}^{2}=.818$. Second, female simulations that acquired the avoidance response with an ITI signal extinguished faster when that cue was removed during extinction (Fig. 4A). However, omission of the ITI signal did not enhance the extinction rate for male simulations. This was confirmed by a significant interaction between sex and ITI signal condition, *F*(1, 36) = 19.01, *p* < .001, ${\eta }_{p}^{2}=.346$. Third, male simulations extinguished faster than female simulations. This was not surprising considering males acquired avoidance to a lesser extent than females, and were therefore expected to extinguish faster. Main effects of session, *F*(11, 396) = 116.287, *p* < .001, ${\eta }_{p}^{2}=.764$, and sex, *F*(1, 36) = 213.264, *p* < .001, ${\eta }_{p}^{2}=.856$, were also found. Similar results were observed when the mean latency to press the lever was examined (Fig. 4C)—an alternative measure of avoidance behavior.

Unlike the empirical data, however, the ITI signal enhanced acquisition in the male, but not female simulations. This was confirmed by a significant interaction between sex and ITI signal condition, *F*(1, 36) = 9.029, *p* = .005, ${\eta }_{p}^{2}=.201$. While there was a trend for the ITI signal to enhance acquisition in SD rats, regardless of sex (Beck et al., 2011), this also did not reach significance. The significant interaction in Fig. 4A could be due to a ceiling effect, precluding observing the effect of the ITI signal in female simulations. Faster acquisition of avoidance associated with an ITI signal is obtained in some studies (Bolles, Grossen & Bond, 1969) but not others (Katzev & Hendersen, 1971), also possibly due to ceiling effects (Galvani & Twitty, 1978). Finally, the discrepancy could also be due to greater variability in the empirical data than in the simulations or a limitation of using only two parameters to simulate sex differences.

Similar to the above, female simulations presented in Fig. 4B acquired the active avoidance response faster than male simulations, and reached higher overall performance. This was confirmed by a significant interaction between session and sex, *F*(4, 145.18) = 29.61, *p* < .001, ${\eta }_{p}^{2}=.451$. There were also main effects of session, *F*(4, 145.18) = 317.255, *p* < .001, ${\eta }_{p}^{2}=.898$, and sex, *F*(1, 36) = 228.857, *p* < .001, ${\eta }_{p}^{2}=.864$. However, these simulations failed to replicate the interaction between sex and ITI signal condition observed above, despite having an identical acquisition phase. This could be due to differences in the initial values of the weights assigned to each input by the critic and actor modules or the responses chosen by the actor early in training.

Unlike simulations where the ITI signal was never presented in the extinction phase (Fig. 4A), the interaction between sex and ITI signal condition failed to reach significance in simulations where the ITI signal was, instead, always present in extinction (Fig. 4B). Taken together, these results indicate that previous experience with the ITI signal is required for it to have an effect during extinction. Therefore, omission of the signal during extinction is not equivalent to presenting it for the first time during extinction. This result represents a novel prediction of the model that is inconsistent with an interpretation of the signal acting as a general indicator of change in the task, leading to generalization decrement and, therefore, faster extinction. Finally, as before, there was a significant interaction between session and sex, *F*(11, 396) = 8.286, *p* < .001, ${\eta }_{p}^{2}=.187$, indicating that male simulations extinguished faster than female simulations. There were also main effects of session, *F*(11, 396) = 157.948, *p* < .001, ${\eta }_{p}^{2}=.814$, and sex, *F*(1, 36) = 194.589, *p* < .001, ${\eta }_{p}^{2}=.844$.

One explanation of these results is that the ITI signal might acquire the properties of a conditioned reinforcer (Weisman & Litner, 1969; Dinsmoor & Sears, 1973; Fernando et al., 2014). Although the ITI signal was not contingent on avoidance in the current protocol, with training, avoidance comes to dominate the behavior. Therefore, on most trials, the ITI signal would follow avoidance, not shock, and could reinforce the avoidance response. This can account for why the ITI signal could, in some cases, enhance acquisition. If the ITI signal acquires reinforcing properties during acquisition, then its omission during extinction would be comparable to removal of reinforcement after avoidance responses, facilitating extinction as shown in Fig. 4A. In contrast, the ITI signal could not become a conditioned reinforcer if it was first presented in extinction (as in Fig. 4B).

An alternative explanation is that the ITI signal may become a positive occasion setter indicating that the warning signal would be followed by shock. The ITI signal could come to play this role through associations with the warning signal formed early in training, prior to high avoidance responding, when the warning signal was more likely to be followed by shock. This association could then be maintained through performance of the avoidance response if the absence of shock was attributed to that response. Subsequent omission of the ITI signal in the extinction phase would then indicate that the warning signal would not be followed by shock, facilitating extinction. This explanation predicts that the ITI signal should acquire properties similar to the warning signal.

**Figure 5:**
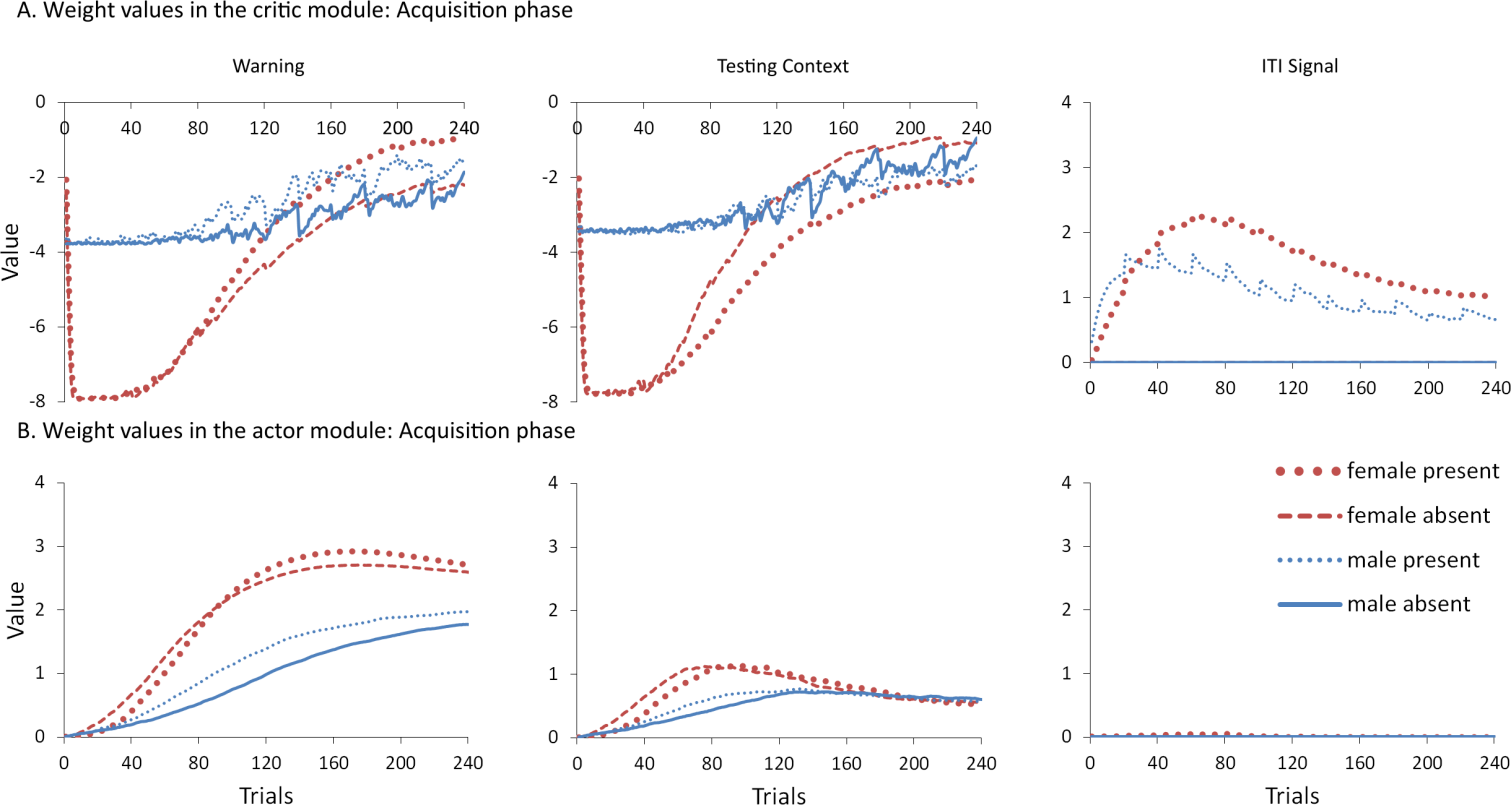
Mean values of the critic (A) and actor (B) weights to inputs representing the warning signal (left), testing context (middle), and ITI signal (right). Weights were recorded at the end of each trial in the acquisition phase for simulations presented in Fig. 4A. The weights in the critic module represent the value assigned to each input stimulus while those in the actor indicate the likelihood of selecting the lever press response when presented with that input. Weights for the home context input were near zero throughout training and are not shown.

To help test these two possible explanations, the mean values of the weights for each stimulus in the critic and actor modules, and how they change with every trial over the course of acquisition, were examined for the simulations presented in Fig. 4A. One set represents the value assigned to each stimulus by the critic component while the other represents the strength of association between each input with all possible actions in the actor component (refer back to Fig. 1). The weights in the actor have the most direct link to what action will be selected, and therefore to the overall behavior of the model. It is important to note that because the probability of selecting a particular action is calculated via an exponential function, even apparently small differences in the weights can have a large impact on behavior. On the other hand, positive and negative values of the weights in the critic module indicate how predictive of reward or punishment, respectively, a given input is Barto (1995). In contrast to the prediction of the occasion setting account, but consistent with the explanation based on conditioned reinforcement, the ITI signal was assigned a positive value by the critic during acquisition (Fig. 5A), suggesting it acquired the properties of a conditioned reinforcer.

In contrast to the positive values assigned to the ITI signal, the values of the inputs representing the warning signal and the testing context were negative in the critic module, first decreasing then reaching an asymptote equal to the value of the shock input, which is determined by the shock cost parameter (Fig. 5A). As described earlier, shock cost is one of the parameters we manipulated in order to simulate male or female-like behavior in the avoidance task. This parameter places a limit on how aversive the warning signal and testing context can become early in training due to their association with shock. As the model acquires the avoidance behavior, the values of the stimuli associated with the shock decrease back toward zero. That is because successful avoidance is never followed by shock, and these stimuli come to represent a less aversive state as avoidance is acquired.

Interestingly, female simulations trained with an ITI signal learned to assign a less negative value to the warning signal and a more negative value to the testing context relative to simulations trained without this signal (Fig. 5A). A similar pattern also occurred for male simulations trained with the ITI signal but the difference was smaller in magnitude. A different picture emerged in the weights to the lever press action in the actor module (Fig. 5B). These weights can only be positive with higher values indicating greater probability that the lever press action will be selected when that input is present. Unlike the weights in the critic, there is a smaller difference for weights in the actor between female simulations trained with and without the ITI signal (Fig. 5B). While the critic module of the female simulations trained with an ITI signal assigned a greater negative value to the testing context than to the warning signal, the actor did the opposite. That is, a lever press response was more likely to be chosen if presented with the warning signal instead of the testing context. Thus, the weights to the warning signal in the critic and actor during acquisition were dissociated. Similarly, the actor and critic also appeared to treat the ITI signal differently. While the ITI signal acquired a positive value in the critic that first increased but then decreased over the acquisition phase of training, the actor assigned little if any weight to this input. Thus, the ITI signal alone should not have the capacity to elicit a lever press response.

Nonetheless, the ITI signal is critical as it appears to modulate the weights assigned to the warning and testing context inputs by both the critic and the actor, and does so to a greater extent in the female simulations. This occurs at least in part because the learning rate parameter that determines the rate of change of the weights in the critic is, by definition, lower for female simulations. The most notable difference in the weights of the actor module is that females have a greater probability to select the lever press action than males, explaining why female simulations acquire avoidance faster than males. By the end of acquisition, male and female simulations appear to have similar weights in the actor irrespective of ITI condition.

**Figure 6:**
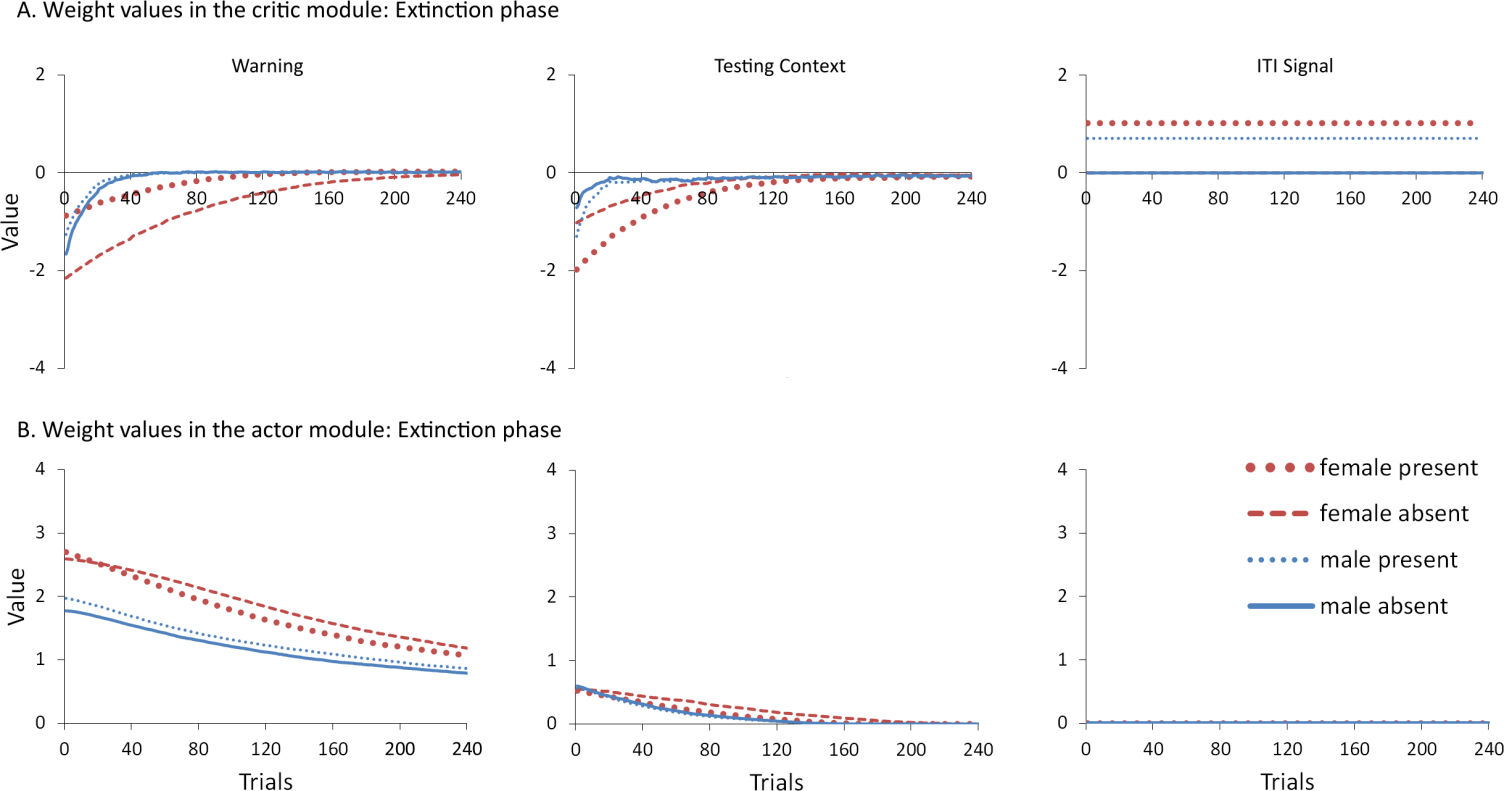
Mean values of the critic (A) and actor (B) weights at the end of each trial in the extinction phase for simulations presented in Fig. 4A. The weights in the actor represent the likelihood of selecting the lever press response when presented with that input. As in acquisition, weights for the home context input were near zero throughout training and are not shown.

However, moving into extinction when the ITI signal is omitted, the weights in the actor for female simulations trained with the signal in acquisition return to zero faster relative to female simulations trained without that signal (Fig. 6B). Because these weights were similar at the end of acquisition (Fig. 5B), this change can be attributed to the final values of the weights in the critic (those by the end of acquisition), and the changes they underwent in extinction. In particular, the value assigned to the warning input in the critic for female simulations trained with the ITI signal was both smaller at the end of acquisition (Fig. 5A) and was the fastest to return to zero in extinction (Fig. 6A). Since the values assigned by the critic are used to adjust the probabilities of actions, this could explain why female simulations trained with the ITI signal moved on to extinguish faster than female simulations trained without the signal, similar to the corresponding groups in Beck et al. (2011). While the ITI signal was also associated with changes in the weights for the testing context, the actor assigned less weight to that input than to the warning (Fig. 6B). Thus, changes in the actor weights for the testing context during extinction likely had less impact on behavior because their values were already near zero.

## Experiment 2: Further Exploration of Parameters in the Model

Experiment 1 showed that faster extinction after omission of the ITI signal in the model can be explained by modulation of the value assigned to the warning signal in the critic by the end of acquisition. The ITI signal appears to have had a greater impact on female simulations due to a lower learning rate in the critic, accounting for the interaction between sex and ITI signal condition. This raises the possibility that it is specifically changes in learning rate in the critic, rather than changes in shock value, that are driving the sex differences. To test this idea, we next conducted simulations where only one of these two values differed.

### Modeling methods

Simulations were trained following the avoidance protocol described above and assigned to ITI signal present or absent conditions. However, instead of considering what we defined earlier as “male” and “female,” we examined the effect of changing only the shock value (−4 vs. −8), holding the learning rate in the critic constant at either 0.01 or 0.05. We also examined the effect of changing only the learning rate in the critic (0.01 or 0.05), with shock value held constant at −4 or −8. The ITI signal was never presented in extinction.

### Results and discussion

For simulations where the learning rate in the critic module (*α*) was held constant (at either 0.01 or 0.05), separate mixed-design ANOVAs with a within-subjects factor of training session, and between-subjects factors of shock cost (−4 or −8) and ITI signal condition (present or absent), were performed on the proportion of avoidance responses. Similar analyses were performed on simulations where shock cost was held constant (at either −4 or −8), in which case *α* (0.01 or 0.05) was a between-subjects factor. In either case, the acquisition and extinction phase were also analyzed separately. For a complete summary of the statistics for these simulations, refer to the Supplemental Information 2, Section 2.

**Figure 7:**
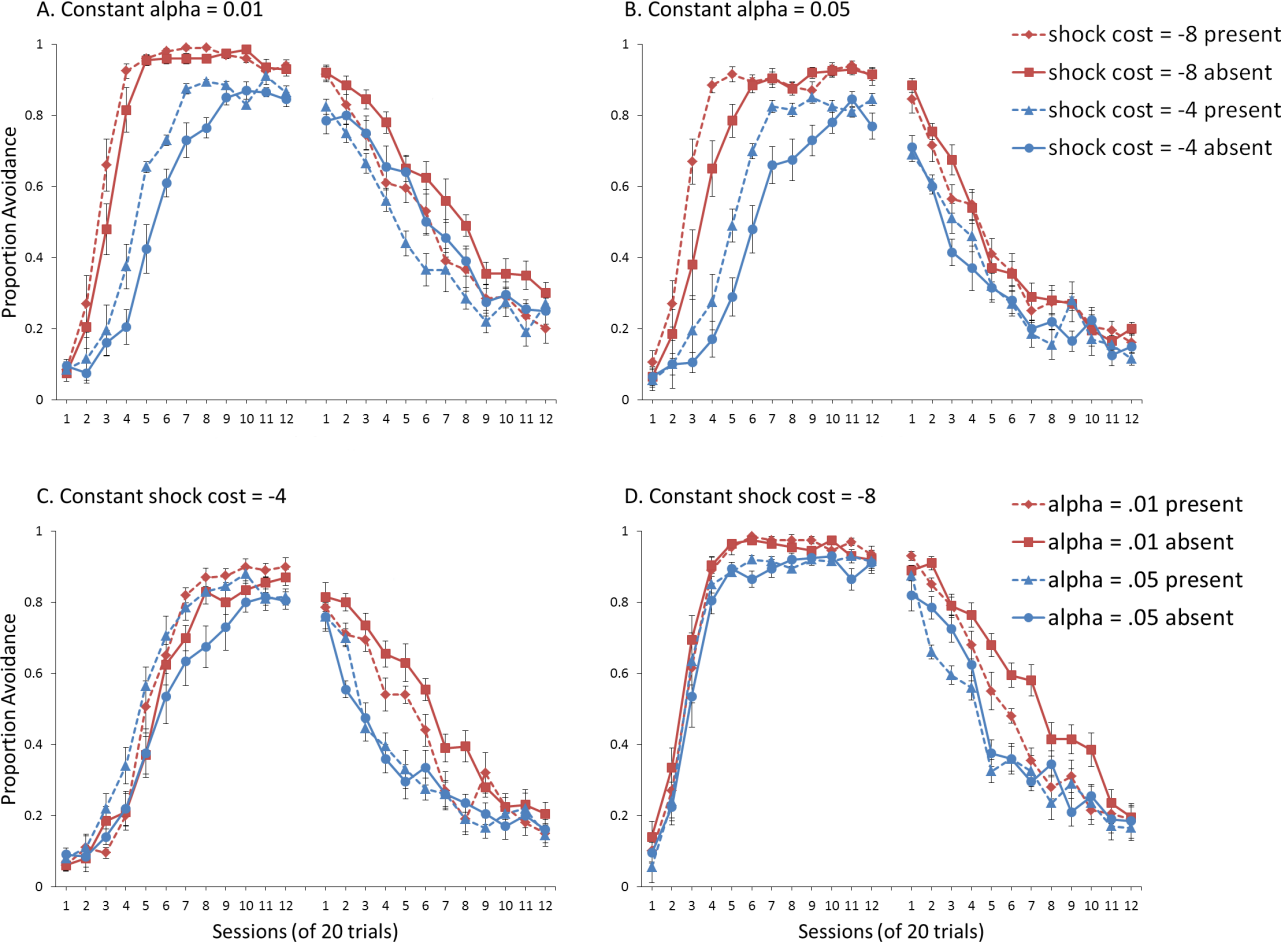
Avoidance behavior for simulations in Experiment 2. Mean proportion avoidance responses for simulations (*n* = 10 per group) changing either (A, B) shock cost (set to −4 for males and −8 for females) or (C, D) the learning rate in the critic module (*α*, set to 0.05 for males and 0.01 for females). For (A) and (B), the learning rate was held constant at 0.01 and 0.05, respectively. For (C) and (D), shock cost was either −4 or −8. An ITI signal was present or absent during acquisition but never presented during extinction.

Simulations where sex differences were defined only by shock cost and *α* was held at 0.01 are presented in Fig. 7A. There was a significant interaction between session and shock cost, *F*(4.2, 152) = 28.434, *p* < .001, ${\eta }_{p}^{2}=.441$, confirming that simulations with higher shock cost acquired avoidance faster and to a greater extent than simulations with lower shock cost. The main effects of session, *F*(4.2, 152) = 347.208, *p* < .001, ${\eta }_{p}^{2}=.906$, and shock cost, *F*(1, 36) = 140.556, *p* < .001, ${\eta }_{p}^{2}=.796$, were also significant. Similar results were obtained for simulations where sex differences were defined by shock cost but *α* was held at 0.05 (Fig. 7B). Again, an interaction between session and shock cost, *F*(4.94, 177.67) = 22.434, *p* < .001, ${\eta }_{p}^{2}=.384$, and main effects of session, *F*(4.94, 177.67) = 279.428, *p* < .001, ${\eta }_{p}^{2}=.886$, and shock cost, *F*(1, 36) = 91.3, *p* < .001, ${\eta }_{p}^{2}=.717$, were found. Taken together, the results suggest shock cost had similar effects on acquisition irrespective of the value of *α*, with higher values leading to both faster acquisition and higher levels of avoidance.

Simulations where sex differences were defined only by the value of *α*, and shock cost was held at −4 are presented in Fig. 7C. There was a significant interaction between session and *α*, *F*(5.6, 200) = 2.207, *p* = .048, ${\eta }_{p}^{2}=.058$, suggesting that higher learning rates in the critic were associated with slower acquisition, in particular near the end of the acquisition phase. Simulations where shock cost was held at −8 (Fig. 7D), only showed a significant main effect of *α*, *F*(1, 36) = 14.711, *p* < .001, ${\eta }_{p}^{2}=.290$, but no interaction with session, indicating that lower learning rates were associated with higher levels of avoidance. Importantly, the size of the effect was smaller than that of the shock cost parameter. Taken together, these simulations suggest modulation of acquisition by the learning rate parameter was both smaller in magnitude and opposite in direction compared to that of the shock cost parameter.

For extinction, the results indicate that simulations where sex differences were defined by shock cost while *α* was held at 0.01 (Fig. 7A) lacked the interaction between shock cost and ITI signal. The signal, however, still enhanced extinction, confirmed by a significant main effect of ITI signal condition, *F*(1, 36) = 25.481, *p* < .001, ${\eta }_{p}^{2}=.414$. This effect was subsequently eliminated when *α* was held at 0.05 (Fig. 7B). Together, these results suggest that changes in shock cost alone, irrespective of the value of the learning rate in the critic module, are insufficient to simulate the empirical data, where omission of the ITI signal enhanced extinction in female, but not male, SD rats.

The ITI signal tended to enhance extinction for simulations where sex differences were defined only by the value of *α*, while shock cost was held at −4 (Fig. 7C). However, this was only at the lower value of *α*, confirmed by a significant interaction between *α* and ITI signal condition, *F*(1, 36) = 8.382, *p* = .006, ${\eta }_{p}^{2}=.189$. The main effect of ITI signal condition, *F*(1, 36) = 5.796, *p* = .021, ${\eta }_{p}^{2}=.139$, was also significant. While this suggests that manipulations of shock cost are not necessary to simulate this interaction, it is important to note that the corresponding acquisition phase did not provide a good fit to the empirical data, where female SD rats acquired avoidance faster than male SD rats (Beck et al., 2011; also see Catuzzi & Beck, 2014). The main effect of sex (as defined by *α*) on acquisition was not significant in these simulations (Fig. 7C), indicating that manipulations of this parameter alone are unable to simulate the observed sex differences.

Similar results were obtained in extinction for simulations where shock cost was held at −8 (Fig. 7D). However, the interaction between *α* and the ITI signal also depended on session, *F*(11, 396) = 2.322, *p* = .009, ${\eta }_{p}^{2}=.061$, suggesting that omission of the ITI signal could only facilitate extinction at the lower *α* level, in particular later in training (sessions 4–10). In contrast, at the higher *α* level, omission of the signal only appeared to enhance extinction early in training (sessions 2–3). These results once again suggest that manipulations of *α* alone are unable to simulate the observed sex differences. Although *α* did modulate acquisition in these simulations (Fig. 7D), those with the higher *α* level (corresponding to males) acquired avoidance much faster and to a greater extent (90% avoidance) than male SD rats, who only reached approximately 70% avoidance (Beck et al., 2011). Thus, these simulations also did not provide a good fit to the empirical data.

Overall, the results suggest changes in *α* level are only sufficient to account for sex differences in ITI signal effects on extinction. In contrast, manipulations of shock cost are only sufficient to simulate sex differences in acquisition rate. Therefore, changes in both parameters are required to simulate the pattern of avoidance learning observed in SD rats.

## Experiment 3: Role of the Testing Context

Experiment 1 found that the critic module of simulations trained with an ITI signal learned to assign a less negative value to the warning signal and a more negative value to the testing context by the end of acquisition. Thus, in addition to the warning signal, the testing context could be important for the effect of the ITI signal. Both the warning signal and ITI signal are associated with this context, providing for an indirect link between them. Thus, if removal of the ITI signal speeds up extinction because of the testing context and its impact on response selection by the actor, then this effect should be eliminated if extinction takes place in a novel context. To test this possibility, we examined avoidance behavior when extinction took place in a new context. That is, the input that represented the testing context in acquisition was set to zero, while a new input served as the context in extinction.

### Modeling methods

Again, male and female simulations were trained with an ITI signal present or absent in acquisition. The ITI signal was never presented during extinction. All conditions received a context shift between acquisition and extinction.

### Results and discussion

A mixed-design ANOVA with within-subjects factor of training session, and between-subjects factors of sex (male or female) and ITI signal condition (present or absent), was performed on the proportion of avoidance responses. The acquisition and extinction phases were analyzed separately. For a complete summary of the statistics, refer to the Supplemental Information 2, Section 3.

**Figure 8:**
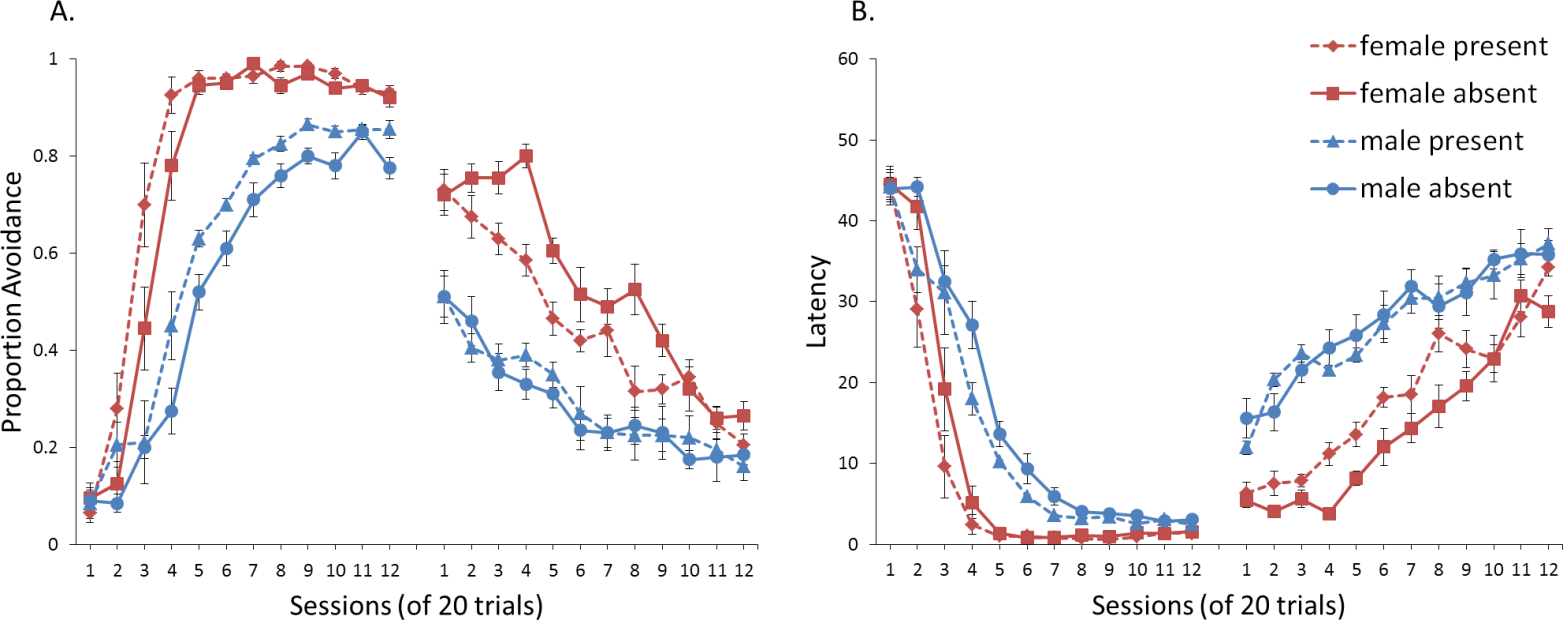
Avoidance behavior for simulations in Experiment 3. Mean (A) proportion avoidance responses and (B) latency to press the lever for simulations (*n* = 10 per group) shifted to a novel training context in extinction. The ITI signal was either present or absent during acquisition but never presented during extinction. As in Experiment 1, Fig. 4A, there was a significant interaction between sex and the ITI signal in the extinction phase, indicating that omission of the ITI signal enhanced extinction in female, but not male, simulations. Therefore, the effect of the ITI signal did not depend on the context.

Results are shown in Fig. 8. As expected, acquisition (before the context shift) was similar to that observed in Experiment 1. Unlike the simulations presented in Fig. 4A, but similar to those in Fig. 4B, the interaction between sex and ITI signal condition was not significant. Instead, the ITI signal was associated with faster acquisition, confirmed by a significant main effect of ITI signal condition, *F*(1, 36) = 16.757, *p* < .001, ${\eta }_{p}^{2}=.318$, suggesting the ITI signal may have acquired conditioned reinforcing properties. As discussed earlier, there was a similar trend in SD rats that did not reach significance (Beck et al., 2011), possibly due to greater variability in the empirical data than in the simulations. Because the acquisition phase in this experiment was identical to that of Experiment 1, the discrepancies between the simulations could once again be due to differences in the initial values of the weights assigned to each input by the critic and actor modules or the responses chosen by the actor early in training. They may also reflect a limitation of using only two parameters to simulate sex differences in the model. Similar to Experiment 1, female simulations acquired avoidance faster than male simulations, indicated by a significant main effect of sex, *F*(1, 36) = 185.07, *p* < .001, ${\eta }_{p}^{2}=.837$.

Finally, there was a visible drop in avoidance for all groups as a result of the context shift between the end of acquisition and the start of extinction. Importantly, despite the context shift, omission of the ITI signal still facilitated extinction in female (but not male) simulations that were trained with that signal during acquisition. This was confirmed by a significant interaction between sex and ITI signal condition, *F*(1, 36) = 11.536, *p* = .002, ${\eta }_{p}^{2}=.243$. Significant main effects of session, *F*(11, 396) = 56.898, *p* < .001, ${\eta }_{p}^{2}=.612$, and sex, *F*(1, 36) = 196.248, *p* < .001, ${\eta }_{p}^{2}=.845$, were also found. These results suggest that omission of the ITI signal does not speed up extinction simply due to associations with the testing context and its impact on response selection by the actor module. Similar results were found when the mean latency to press the lever was examined (Fig. 8B).

## Experiment 4: ITI Duration

The duration of the ITI may also be important for the effect of the ITI signal (Berger & Brush, 1975; Galvani & Twitty, 1978). For example, a shorter ITI (and correspondingly shorter ITI signal) results in less experience with, and opportunity to learn about the ITI signal. Reducing the interval between shocks could also make the ITI signal more aversive, possibly reducing or even reversing its effects on avoidance. Finally, a shorter ITI also reduces the opportunity for unreinforced lever press responses (inter-trial responses, ITRs), potentially decreasing the likelihood that lever presses in the presence of the ITI signal are not reinforced. In this experiment, we examined the effect of omitting the ITI signal during extinction when ITI duration was shortened.

### Modeling methods

Simulations (male and female, ITI signal present or absent) were trained with an ITI length of 30 s (reduced from 180 s). All other model parameters and the training procedure were the same as described in the general method. As in the previous simulations, as well as in the experimental protocol of Beck et al. (2011), the duration of the ITI was equal to that of the ITI signal.

### Results and discussion

A mixed-design ANOVA identical to that of Experiment 4 was conducted on the proportion of avoidance responses. The acquisition and extinction phases were again analyzed separately. For a complete summary of the statistics, refer to the Supplemental Information 2, Section 4.

**Figure 9:**
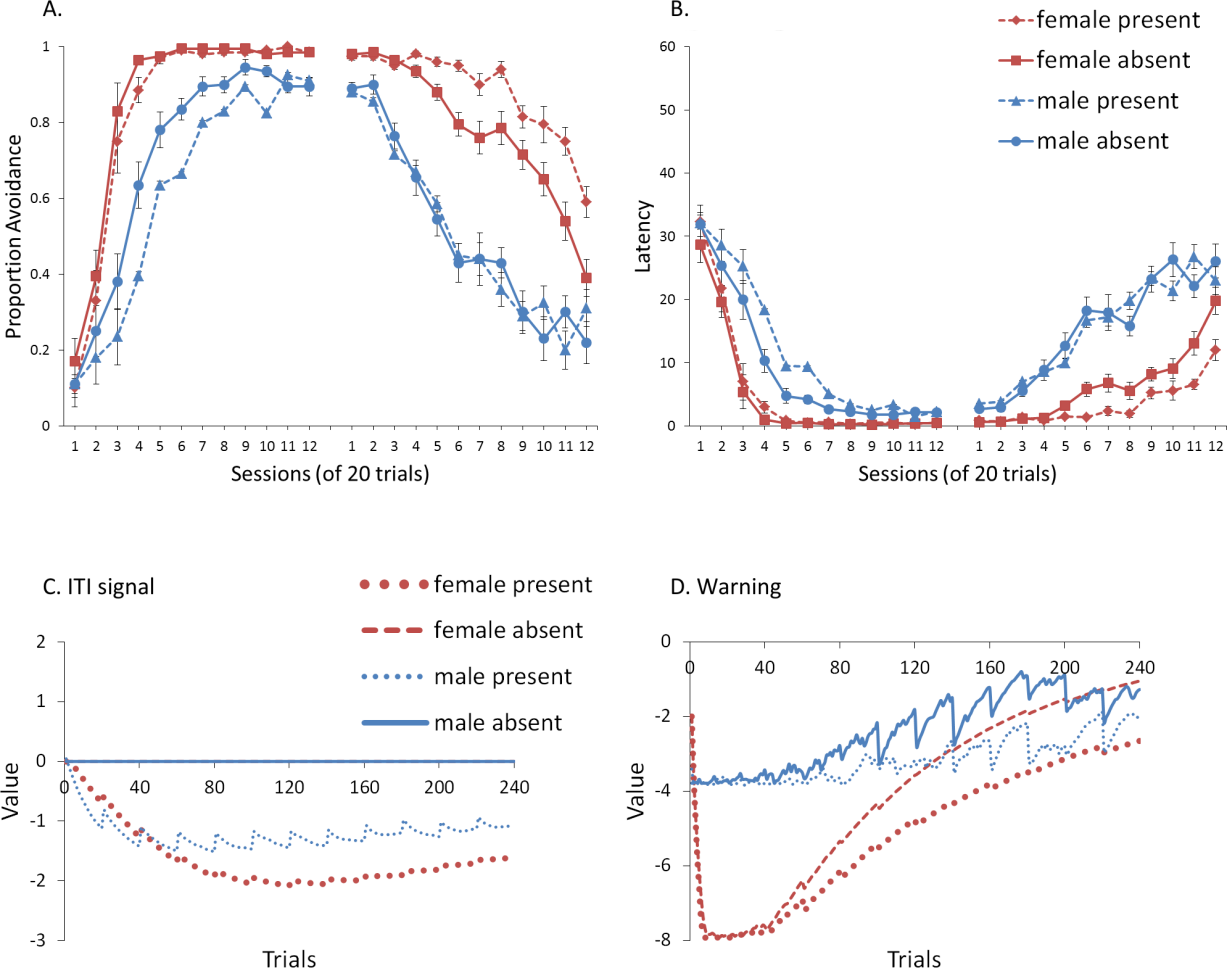
Simulations (*n* = 10 per group) trained with an ITI length of 30 s. Mean (A) proportion avoidance responses and (B) latency to press the lever with the value of the weights assigned to the (C) ITI signal and the (D) warning signal by the critic module of the model in the acquisition phase. An ITI signal was either present or absent during acquisition, but never presented during extinction.

Interestingly, when ITI duration was shortened, the effect of the ITI signal was reversed (relative to Experiment 1, Fig. 4A) for both acquisition and extinction. The signal now tended to slow (rather than enhance) acquisition. This was confirmed by a significant main effect of ITI signal condition, *F*(1, 36) = 12.108, *p* = .001, ${\eta }_{p}^{2}=.252$. As before, significant main effects of session, *F*(4.39, 158.15) = 248.148, *p* < .001, ${\eta }_{p}^{2}=.873$, and sex, *F*(1, 36) = 123.247, *p* < .001, ${\eta }_{p}^{2}=.774$, were also found. Omission of the ITI signal now slowed extinction in female, but not male, simulations trained with the signal during acquisition (Fig. 9A). This was confirmed by a significant interaction between sex and ITI signal condition, *F*(1, 36) = 12.723, *p* < .001, ${\eta }_{p}^{2}=.261$. There were also significant main effects of sex, *F*(1, 36) = 512.523, *p* < .001, ${\eta }_{p}^{2}=.934$, and ITI signal, *F*(1, 36) = 11.705, *p* = .002, ${\eta }_{p}^{2}=.245$. Similar results were found when the mean latency to press the lever was examined (Fig. 9B).

These results suggest that the removal of the ITI signal does not always speed up acquisition or extinction, and that its effect depends on features of the task related to the ITI. In contrast to simulations where omission of the ITI signal enhanced acquisition, in which the ITI signal was assigned positive values by the critic module (Fig. 5A), here the signal was assigned negative values (Fig. 9C). Therefore, the ITI signal may have acquired aversive properties allowing it to serve as a negative reinforcer of the avoidance behavior. Its subsequent omission may have reinforced avoidance, and led to resistance to extinction. As discussed earlier, the results of the previous simulations could be interpreted in terms of a positive occasion setting account, but there were difficulties with that explanation given that the ITI signal acquired a positive value in the critic. The current simulations pose further difficulties for that explanation. While the ITI signal was assigned a negative value (Fig. 9C) similar to the warning (Fig. 9D) as would be predicted if it was a positive occasion setter, the signal’s removal instead slowed extinction, inconsistent with that account.

Alternatively, it could be assumed that the ITI signal became a negative occasion setter given that once avoidance responding is high, presentation of the ITI signal will be followed by the warning, absent of shock. Previous studies have suggested that the avoidance response itself comes to play a similar role (De Houwer, Crombez & Baeyens, 2005), but see (Declercq & De Houwer, 2011). Omission of the negative occasion setter could then increase avoidance, as observed here. However, if the ITI signal had become a negative occasion setter, it should have been assigned a positive value in the critic because it would predict the absence of an aversive state. Instead it acquired a negative value, inconsistent with this account. Once again, an explanation based on conditioned reinforcement appears more likely.

**Figure 10:**
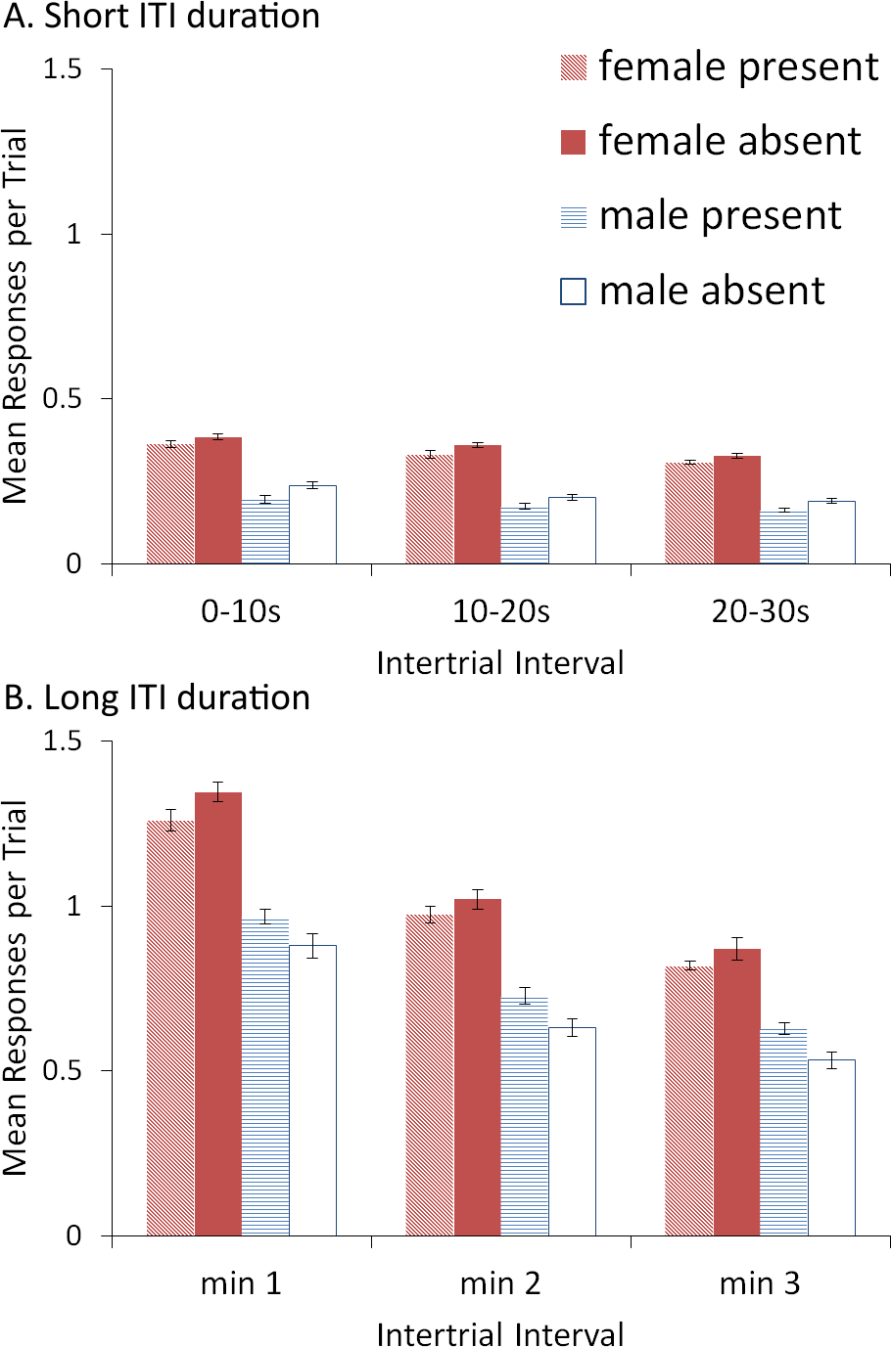
ITRs for simulations trained with an ITI length of 30 or 180 s. ITRs for simulations (*n* = 10 per group) trained with an ITI length of 30 s split into 10 s segments (A) and simulations trained with ITI length of 180 s split into 1 min segments (B); corresponding avoidance behavior is shown in Figs. 9 and 4, respectively. An ITI signal was either present or absent in acquisition, but was never presented during extinction.

The shorter ITI duration also reduced the opportunity for ITRs (Fig. 10A). ITRs may be one reason why omission of the signal modulates extinction as they can change the values of the weights acquired by both the critic and actor components of the model. It is important to note that when the length of the ITI was 180 s (Fig. 10B), the ITRs decreased across each minute of the ITI, a pattern also observed in the empirical data (Beck et al., 2011). This was confirmed by a significant main effect of ITI segment, *F*(2, 72) = 422.958, *p* < .001, ${\eta }_{p}^{2}=.922$. The model predicted that a similar pattern should be observed with shorter ITI duration (Fig. 10A), also confirmed by a significant main effect of ITI segment, *F*(2, 72) = 44.404, *p* < .001, ${\eta }_{p}^{2}=.552$, though the effect was notably smaller. Additionally, female simulations made more ITRs than male simulations—a trend also present in rats (Beck et al., 2011). The model predicted that this should be independent of ITI duration, confirmed by significant main effects of sex for simulations with an ITI duration of 30 s, *F*(1, 36) = 465.231, *p* < .001, ${\eta }_{p}^{2}=.928$, and 180 s, *F*(1, 36) = 213.928, *p* < .001, ${\eta }_{p}^{2}=.856$. Finally, when ITI duration was shorter (Fig. 10A), the ITI signal tended to reduce ITRs, confirmed by a significant main effect of ITI signal condition, *F*(1, 36) = 15.634, *p* < .001, ${\eta }_{p}^{2}=.303$. In contrast, when ITI duration was longer, the ITI signal reduced ITRs in female (but not male) simulations (Fig. 10B), suggested by a significant interaction between sex and ITI signal condition, *F*(1, 36) = 12.294, *p* = .001, ${\eta }_{p}^{2}=.255$. This was confirmed via Bonferroni corrected independent samples *t*-tests performed on ITRs averaged across the ITI, which only found a significant difference between female simulations trained with and without the signal, *t*(18) = − 3.044, *p* = .007, *r* = 0.58.

## Experiment 5: Role of ITRs

Results from Experiment 4 suggest that ITRs may be important for whether omission of the ITI signal will speed up or slow extinction. However, in the prior experiment, duration of the ITI was confounded with ITRs, since longer ITIs provide more opportunity to exhibit ITRs. To better understand the effect of ITRs, another experiment was conducted in the model where ITI length was held constant at either 180 or 30 s while the lever press response was disabled during the ITI, eliminating the possibility of any ITRs. This manipulation is analogous to retracting the lever during the ITI of a behavioral experiment. It should be noted that retraction of the lever could serve as an ITI signal in and of itself to animals learning the avoidance task but that the state of the lever was not represented by an input in the model. Thus, an advantage of using the model is the ability to remove this confound, which may not be completely possible in a behavioral experiment.

### Modeling methods

Simulations were conducted as before (male and female, ITI signal present or absent); within each condition, half of the simulations received an ITI of 180 s and half received an ITI of 30 s. During the ITI in both acquisition and extinction, lever press was “disabled,” meaning that the actor module could not execute that response. This completely eliminated ITRs, since no lever press responses could be executed during the ITI. All other parameters and the training procedure were the same as described in the general method.

### Results and discussion

A mixed-design ANOVA with within-subjects factor of session, and between-subjects factors of sex (male or female), ITI signal condition (present or absent), and ITI duration (30 or 180 s) was performed on the proportion of avoidance responses. The acquisition and extinction phases were analyzed separately. The interaction between session, sex, ITI signal, and ITI duration was significant in analyses of both acquisition, *F*(5.13, 369.66) = 5.518, *p* < .001, ${\eta }_{p}^{2}=.071$, and extinction, *F*(8.09, 582.75) = 2.998, *p* = .003, ${\eta }_{p}^{2}=.040$. To understand these interactions, simulations with different ITI durations were analyzed separately. For a complete summary of statistics, refer to the Supplemental Information 2, Section 5.

**Figure 11:**
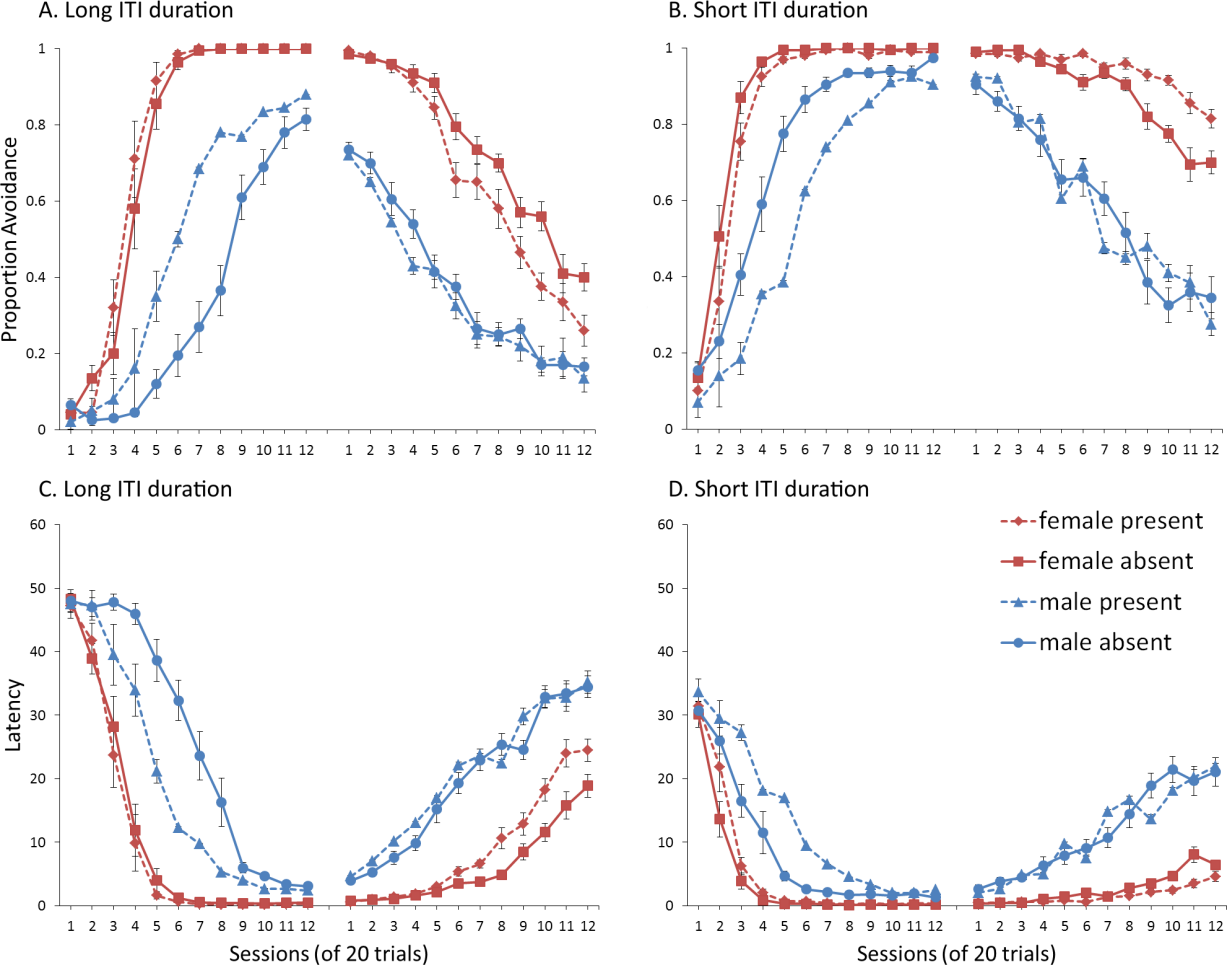
Avoidance behavior for simulations in Experiment 5. Mean proportion avoidance responses (A–B) and latency to press the lever (C–D) for simulations (*n* = 10 per group) trained with an ITI length of 180 (A, C) or 30 s (B, D). An ITI signal was either present or absent during acquisition, but was never presented during extinction.

Although ITRs were completely eliminated in this experiment (data not shown), omission of the ITI signal still facilitated extinction in the female simulations with a 180-sec ITI, although this did not occur until later in the extinction phase (Fig. 11A) relative to simulations in which the lever press response was available (Fig. 4A). This was confirmed by a significant interaction between session, sex and ITI signal, *F*(11, 396) = 1.885, *p* = .040, ${\eta }_{p}^{2}=.050$. The main effect of sex was also significant, *F*(1, 36) = 505.715, *p* < .001, ${\eta }_{p}^{2}=.934$. In Experiment 1, Fig. 4A, when the response was available during the ITI, facilitation was evident by session 3, while in the current simulations it did not appear until session 5, and was most evident starting at session 6 of extinction. This suggests that while the ITRs themselves may not be critical for the effect of the ITI signal, they still modulate it. Similar results were found when the mean latency to press the lever was examined (Fig. 11C).

**Figure 12:**
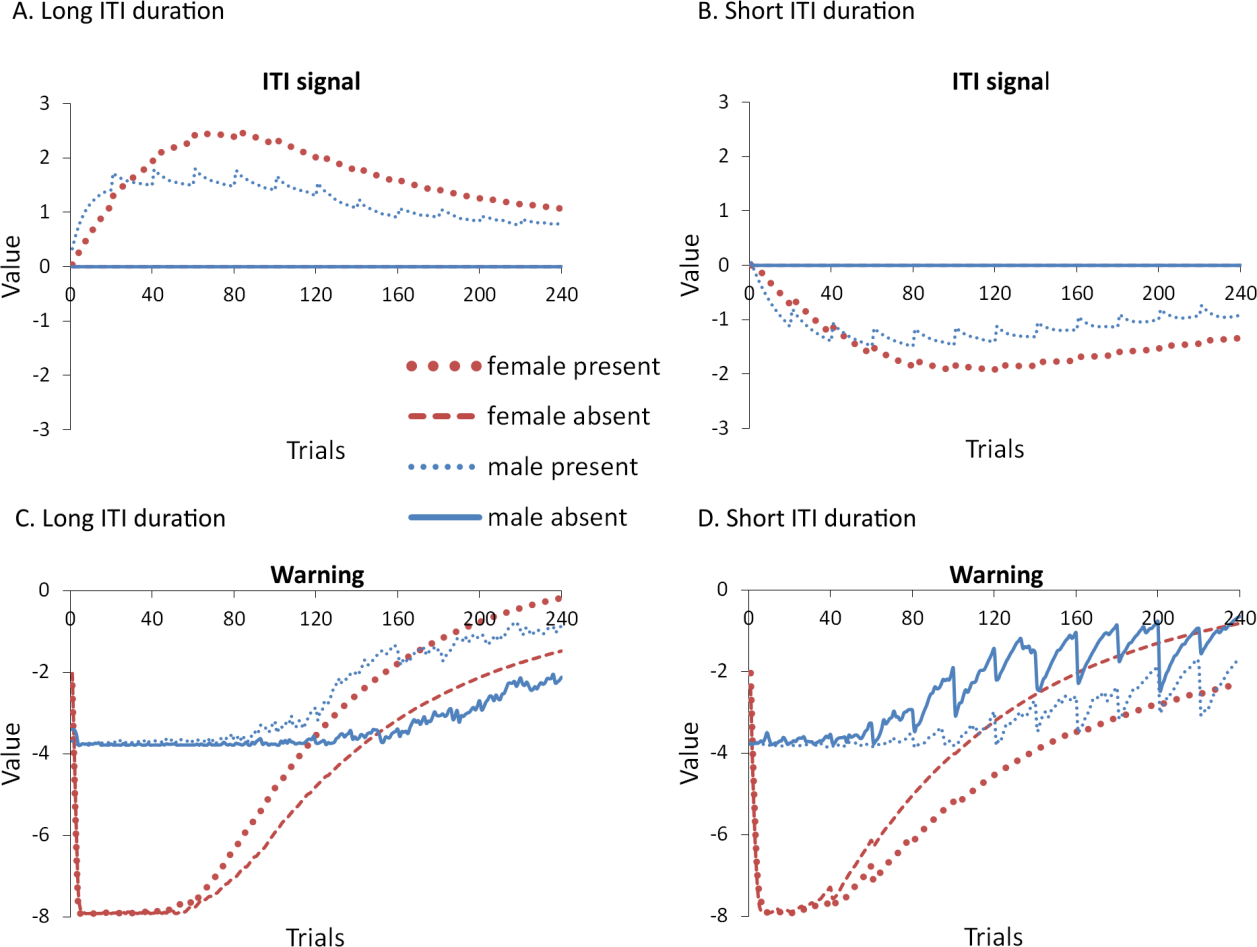
Weights in the critic module for simulations in Experiment 5. Mean values of the weights assigned to the (A, B) ITI signal or the (C, D) warning signal in the critic module of the model during the acquisition phase for simulations trained with an ITI length of 180 (A, C) or 30 s (B, D). Corresponding avoidance behavior is presented in Fig. 11.

The simulations also predict that eliminating the ITRs should alter how the ITI signal is used when ITI duration is short, with similar results for both proportion avoidance responses (Fig. 11B) and latency to press the lever (Fig. 11D). While there was a trend for the effect of the ITI signal to reverse, similar to Experiment 4 where ITI duration was also 30 s but ITRs were allowed (Fig. 9A), in the current simulations, neither the main effect of the ITI signal, nor the interaction between sex and the ITI signal, reached significance, (*p* = .052 and .087, respectively). These trends suggest that omission of the ITI signal may slow extinction in female simulations when the ITI is shorter, but not until later in training, similar to when the length of the ITI is longer. As in Experiment 4, the ITI signal was assigned a positive value in the critic when the ITI was longer and its omission led to faster extinction (Fig. 12A), and was assigned a negative value when the ITI was shorter and it tended to slow extinction (Fig. 12B). Similar to before, the warning also acquired a less negative value (Fig. 12C) or a more negative value (Fig. 12D) in simulations trained with the ITI signal when the ITI was longer or shorter, respectively.

As in some of the previous experiments, the ITI signal facilitated acquisition in male simulations trained with the longer ITI (Fig. 11A), but impaired learning when ITI length was shorter (Fig. 11B). In either case, it had minimal impact on acquisition in female simulations. This was confirmed by a significant interaction between sex and ITI signal condition, *F*(1, 36) = 7.768, *p* = .008, ${\eta }_{p}^{2}=.266$ and *F*(1, 36) = 10.559, *p* = .003, ${\eta }_{p}^{2}=.227$, for simulations with an ITI length of 180 and 30 s, respectively. Note that all possible main effects and all other possible interactions were also significant. Again, this could be explained if the ITI signal was capable of reinforcing or punishing avoidance responding, respectively, consistent with the positive (Fig. 12A) and negative values (Fig. 12B) it was assigned in the critic module. It had little impact on female simulations likely because its effects were masked by the high shock value, which led to very high avoidance rates. Finally, in this experiment, ITI duration was still equal to that of the ITI signal. Thus, the duration of the ITI signal alone, as well as its timing within the ITI, could also be important. The next experiment examined this possibility.

## Experiment 6: ITI Signal Duration and Timing

In our final experiment, we considered the possibility that the duration of the ITI signal, independent of the duration of the ITI, is important for its effects on avoidance. This experiment also tested the possibility that the ITI signal serves as a positive occasion setter for shock. If the ITI signal serves as an occasion setter then presenting the signal earlier during the ITI so that it is not immediately followed by the warning signal should interfere with learning that association. In this case, subsequent omission of the signal should no longer speed up the rate of extinction. To examine this, the length of the ITI was held constant at 180 s while the length of the ITI signal was reduced to 30 s. The signal was then presented either in the first or the last 30 s of the ITI. It is important to note that the effect of the ITI signal may not depend on its absolute duration, but rather on its duration relative to the danger-free period that follows the ITI signal, prior to the next presentation of the warning (Cándido et al., 1991). These simulations control for this possible confound by keeping the duration of the ITI signal and the signal-free portion of the ITI constant. Only the timing of the signal within the interval was varied.

### Modeling methods

**Figure 13:**
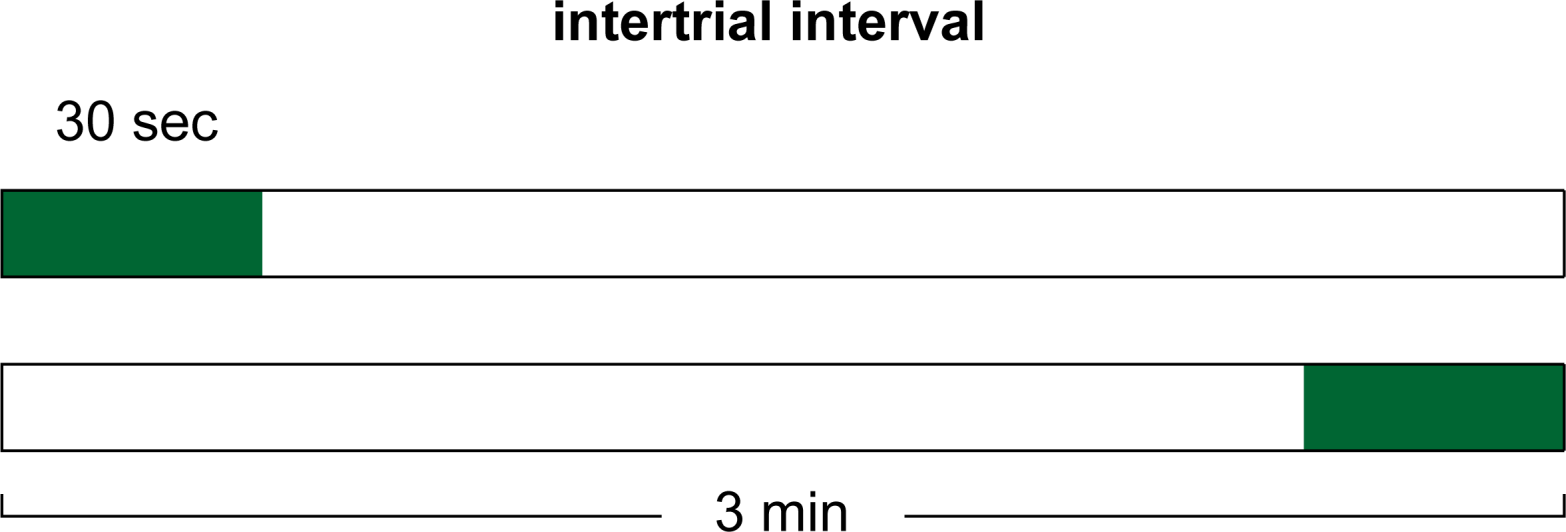
Schematic of the ITI for Experiment 6. An ITI signal could be presented during the first or last 30 s of the 180-sec ITI in the acquisition phase. No ITI signal was presented in the extinction phase for any of the simulations.

Simulations were conducted with male and female parameters. As before, the ITI signal could be present or absent in acquisition, while it was never presented in extinction. For all simulations, the length of the ITI was held constant at 180 s while that of the ITI signal was reduced to 30 s. However, for half of the simulations the ITI signal appeared at the beginning of the ITI, and for half it appeared at the end, as illustrated in Fig. 13. All other aspects of the training procedure were identical to those in the general method.

### Results and discussion

A mixed-design ANOVA with within-subjects factor of session, and between-subjects factors of sex (male or female), ITI signal condition (present or absent), and ITI signal timing (first or last 30 s of the ITI) was performed on the proportion of avoidance responses. The acquisition and extinction phases were analyzed separately. Female simulations acquired avoidance faster and to a greater extent than male simulations, confirmed by a significant interaction between session and sex, *F*(4.4, 313.8) = 41.342, *p* < .001, ${\eta }_{p}^{2}=.365$, and significant main effect of sex, *F*(1, 72) = 314.747, *p* = .001, ${\eta }_{p}^{2}=.814$. The interaction between sex and ITI timing was also significant, *F*(1, 72) = 4.676, *p* = .034, ${\eta }_{p}^{2}=.061$. In the analysis of extinction, there was a significant interaction between sex, ITI signal condition, and ITI signal timing, *F*(1, 72) = 8.056, *p* = .006, ${\eta }_{p}^{2}=.101$. To understand these interactions, simulations with different ITI signal timing were analyzed separately. Finally, because the interaction between sex, ITI signal condition and ITI signal timing only reached significance in extinction in the analysis of the latency to press the lever, *F*(1, 72) = 6.28, *p* = .014, ${\eta }_{p}^{2}=.080$, only that phase was examined further. For a complete summary of statistics, refer to the Supplemental Information 2, Section 6.

**Figure 14:**
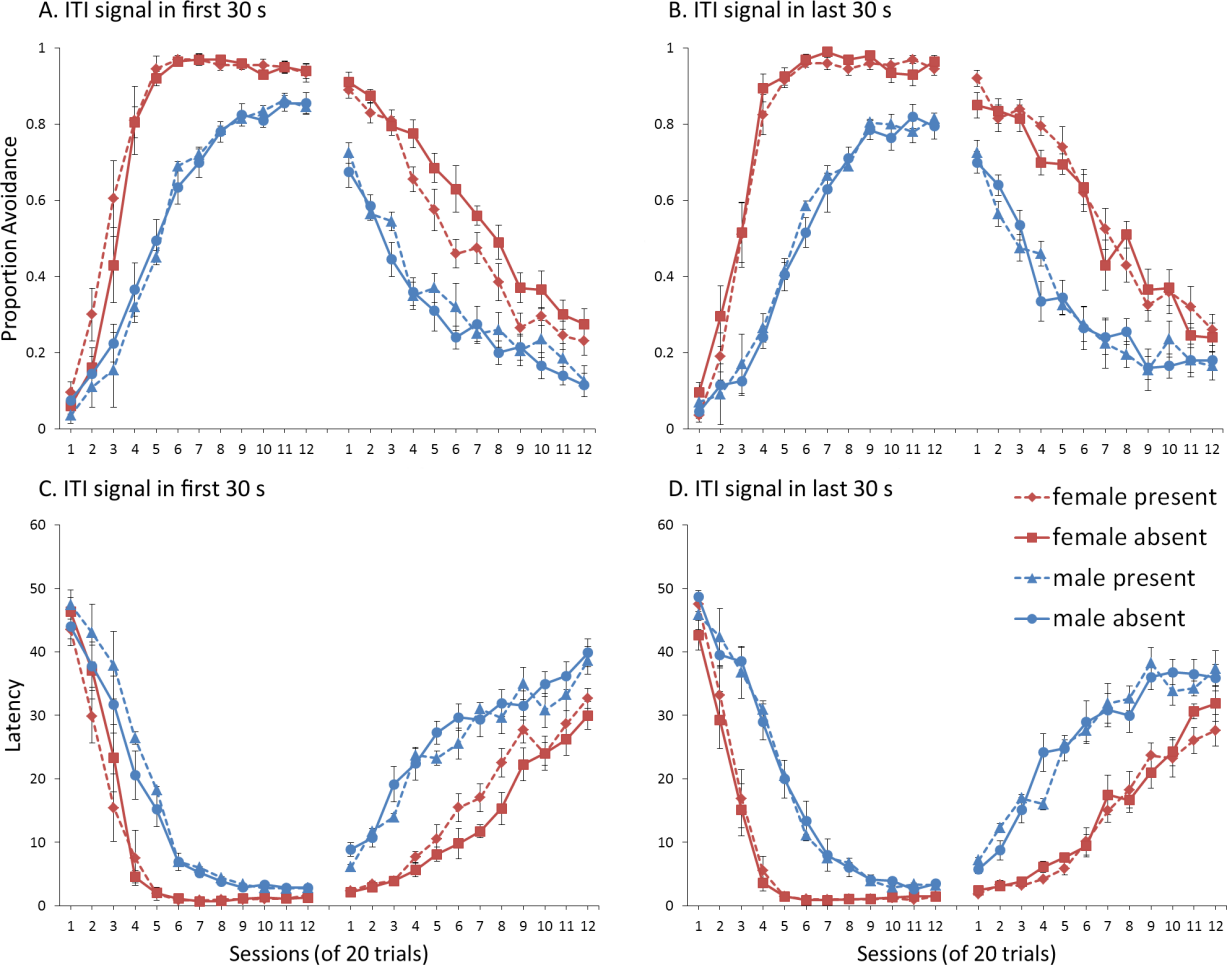
Avoidance behavior for simulations in Experiment 6. Mean proportion avoidance responses (A, B) and latency to press the lever (C, D) for simulations (*n* = 10 per group) trained with an ITI signal in the first (A, C) or last (B, D) 30 s of the ITI. The ITI signal could be present or absent during acquisition, but was never presented during extinction.

The results suggest that the timing of the ITI signal is critical for how its omission will affect extinction. Omission of the ITI signal when it was presented in the first 30 s of the ITI facilitated extinction for female, but not male, simulations (Fig. 14A). This was confirmed by a significant interaction between sex and ITI signal condition, *F*(1, 36) = 12.802, *p* < .001, ${\eta }_{p}^{2}=.262$. In contrast, omission of the ITI signal had no effect on extinction if it was presented in the last 30 s of the ITI (Fig. 14B). These results argue against the occasion setting account. It should have been easier for the ITI signal to become an occasion setter when presented in the last 30 s of the ITI where it would be immediately followed by the warning signal. Under these conditions, the ITI signal’s subsequent omission should have enhanced extinction to a greater extent, or at least as well as, when there was a gap between presentation of the ITI signal and the warning. Instead, omission of the ITI signal only affected extinction when it was presented in the first 30 s of the ITI. It is important to note that prior research has shown that the avoidance response itself could serve as a negative occasion setter (De Houwer, Crombez & Baeyens, 2005). While the role of the avoidance response was not considered here, occasion setting could still be important for avoidance learning. More recently, however, it has been argued that prior studies have not provided unique support for this occasion setting account (Declercq & De Houwer, 2011). The results of the current simulations also argue against an occasion setting account of avoidance learning, a prediction that could be tested by future empirical work.

**Figure 15:**
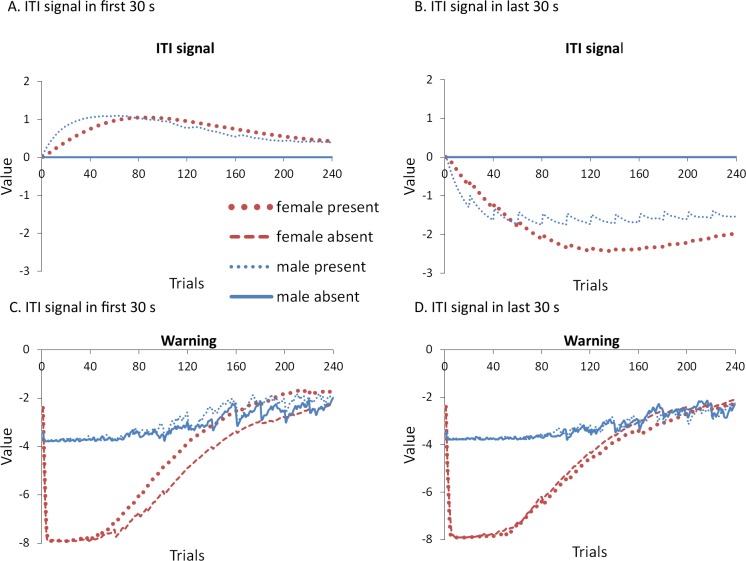
Weights in the critic module for simulations in Experiment 6. Mean values of the weights assigned to the (A, B) ITI signal and the (C, D) warning signal in the critic module at the end of each trial in the acquisition phase when the ITI signal was presented in the first (A, C) or the last (B, D) 30 s of the ITI. Positive and negative values could indicate the ITI signal had acquired appetitive and aversive properties, respectively.

As before, these results can be explained if it is assumed that the ITI signal became a positive reinforcer of the avoidance behavior (Weisman & Litner, 1969; Dinsmoor & Sears, 1973; Fernando et al., 2014). Consistent with this, the value of the ITI signal was positive in the critic module when it was presented in the first 30 s of the ITI (Fig. 15A). This was also when its omission enhanced extinction. In contrast, this value was negative when it was presented in the last 30 s of the ITI (Fig. 15B), when omission had no effect on extinction. As discussed earlier, the values assigned to inputs in the critic module can be thought of as the valence of those inputs. Thus, positive values could indicate that the ITI signal was able to reinforce avoidance responding.

Though the difference was small, when the ITI signal acquired a positive value, this was greater in female compared to male simulations (Fig. 15A). At the same time, the warning signal acquired a less negative value in the simulations trained with the ITI signal (relative to those trained without it), and this difference was larger in the female simulations (Fig. 15C). This suggests that the ITI signal may have been more reinforcing in the female simulations and was, therefore, also more likely to decrease avoidance behavior when omitted in the extinction phase, accounting for the observed sex difference in its effect on extinction. Consistent with this interpretation, the signal was also assigned a more positive value by the critic in the female simulations from Experiment 1. Additionally, the sex difference in the value was larger in that experiment (Fig. 5A), which was also coupled with a larger effect on avoidance during extinction (Fig. 4A) than that observed here.

In either case, when the ITI signal acquired positive values, the warning signal acquired less negative values. This could be because the warning had also become a predictor of what was now, in essence, an appetitive event (presentation of the ITI signal). This could have occurred since avoidance responding is high late in acquisition. Therefore, the warning signal was reliably followed by the ITI signal instead of by shock. In contrast, the negative values assigned to the ITI signal when it was presented in the last 30 s of the ITI (Fig. 15B) suggest that it had acquired aversive properties, similar to the warning signal (and due to its close association with that signal). However, in this case the ITI signal could not modulate avoidance as it did not follow the avoidance response. Likewise, the warning signal acquired similar values in female simulations irrespective of ITI signal condition (Fig. 15D). Finally, it is important to note that the current simulations do not directly test whether the ITI signal acquired the properties of a conditioned reinforcer, which could be confirmed by future work as suggested further below.

## Conclusions

Experiment 1 showed that a reinforcement learning model, previously able to address strain differences in escape-avoidance behavior in male rats, could be extended to account for sex differences as reported by Beck et al. (2011). Specifically, we simulated males vs. females via the manipulation of two parameters—shock cost and the learning rate of the critic module. Experiment 2 showed that these parameters had dissociable effects on avoidance. Changes in the shock cost, representing the aversive value of reinforcement, were necessary to simulate sex differences in acquisition rate. In contrast, changes in the learning rate of the critic module, which represents how quickly the model revises its expectations about future reinforcement, were necessary to simulate sex differences in ITI signal use. Together, changes in these parameters were able to simulate the experimental observation that the omission of a signal previously associated with a safe period between presentations of an aversive event (i.e., during the ITI) will facilitate the extinction of avoidance behavior in female but not male SD rats (Beck et al., 2011; Catuzzi & Beck, 2014).

The simulations also made several novel predictions, specifically, that whether omission of an expected ITI signal can enhance the rate of extinction in females, but not males, should depend on the duration of the ITI, the duration of the signal itself, and its timing within the interval. When signal onset was delayed until later in the ITI, removal of the signal no longer affected extinction in female simulations, suggesting that the signal does not serve as an occasion setter, and instead, could serve as a conditioned reinforcer of the avoidance behavior. Non-reinforced responses during the ITI were also found to modulate how the signal was used. In the absence of ITRs, removal of the ITI signal still facilitated extinction in female simulations but this effect was delayed until later in extinction training. Overall, the results suggest that a computational model that embodies mechanisms from several theories of avoidance learning, including two-factor theory (Maia, 2010; Mowrer, 1951), can account for individual differences in the use of signals associated with periods of safety.

In addition to testing the predictions of the model about escape-avoidance behavior, future work could test the model’s prediction that the ITI signal acquires the properties of a secondary reinforcer. For instance, Fernando et al. (2014) found that male rats preferred to press a lever that both avoided shock and led to the presentation of an ITI signal, relative to a lever that only avoided shock. Future work could extend the current model to simulate a similar paradigm, and examine if there are any sex differences as a function of the ITI signal. It is important to note that in several prior studies (Bolles, Grossen & Bond, 1969; Katzev & Hendersen, 1971), including those that have examined the reinforcing properties of ITI (or safety) signals (Weisman & Litner, 1969; Dinsmoor & Sears, 1973; Fernando et al., 2014), the ITI signal was contingent on successful avoidance and is not present after escape or shock trials as it was here. Therefore, whether or not the ITI signal is contingent on avoidance behavior could be an important factor to consider in future research.

Katzev & Hendersen (1971) found that an ITI signal contingent on avoidance only protected from extinction if it provided new information during acquisition, that is, only if warning termination was delayed until a period of time after the avoidance response. In the avoidance learning paradigm simulated here, both the offset of the warning and the onset of the ITI signal should provide the same information since both occur when the lever is pressed. While Katzev & Hendersen (1971) failed to find protection from extinction under these conditions, they only used male rats. Thus, their results are not at odds with the current simulations because male simulations also failed to show protection from extinction as long as the parameter governing the learning rate in the critic module was set high enough. Less clear, however, is the role of an ITI signal that is contingent on avoidance. Future simulations can confirm if the same model will make different predictions about the effect of the ITI signal if it is contingent on successful avoidance as opposed to always present during the ITI. However, it is important to note that the two versions of the avoidance paradigm should converge later in training as avoidance is acquired because most of the trials would in fact be successful avoidance trials. Thus, later in training, the ITI signal will follow, by and large, only avoidance responses. Nonetheless, experience with the signal early in training could still alter how that signal is used later.

Future work could also simulate strain differences in ITI signal use. As discussed earlier, WKY rats, an animal model of anxiety vulnerability, differ in how they learn to avoid aversive events relative to SD rats (Servatius et al., 2008). Prior simulations have already shown that the current model can account for these strain differences in avoidance learning, including faster acquisition, resistance to extinction and the lack of warm-up between training sessions in the WKY rats (Myers et al., 2014). In the same lever press avoidance paradigm, male WKY rats were also found to acquire avoidance faster in the presence of an ITI signal, unlike male and female SD rats (Beck et al., 2011). Similar to male SD rats, omission of the ITI signal did not speed up extinction in male WKY rats, but WKY rats extinguished slower than SD rats irrespective of the ITI signal (Beck et al., 2011). Future simulations can determine if the model can also explain these strain differences in the use of the ITI signal. It is important to note that, in some cases, the current simulations predicted that males should acquire avoidance faster in the presence of the ITI signal, at odds with male SD but similar to male WKY behavior (Beck et al., 2011). However, past simulations of male SD behavior (Myers et al., 2014) via the same parameters as those used to simulate males in the current study do show warm-up, which is not found in the WKY strain (Beck et al., 2011; Servatius et al., 2008). This suggests that additional parameter manipulations may be needed to fully account for the observed strain differences in ITI signal processing. Some studies have reported faster (i.e., supernormal) acquisition in the presence of ITI signals in a variety of avoidance paradigms (Bolles, Grossen & Bond, 1969). Thus, more research is also needed to clarify the conditions under which the ITI signal can facilitate acquisition of avoidance.

Sex differences also exist in how ITI signals affect human approach-avoidance learning. Sheynin et al. (2014) found that the presence of an ITI signal impaired acquisition while its omission facilitated extinction in female participants. A computational model similar to the one used here was able to simulate these sex differences (Sheynin et al., 2015). As in Beck et al. (2011) and the simulations presented here, the ITI signal in that study was non-contingent on behavior. However, Sheynin et al. (2014) used a task with competing approach and avoidance components. Participants could either shoot a spaceship to earn points or avoid a bomb in order to prevent loss of points, but could not earn points while avoiding. Future studies could examine if ITI signals also affect human escape-avoidance learning and determine if the same model that was able to explain the key features of rodent escape-avoidance can also account for human behavior in that task. Sheynin et al. (2014) also used a cognitive aversive event (i.e., point loss) while rodent studies use physical aversive stimuli (i.e., shock). The current model simulated male vs. female avoidance behavior, in part, based on sex differences in pain sensitivity in rodents. Potential differences between cognitive and physical aversive outcomes in human avoidance learning, which could vary as a function of sex, are also important to consider in future research.

There are several key limitations of the current work, of which the most important is an obvious simplification of sex differences to the manipulation of two parameters in a fairly simple learning model. Clearly, sex differences in rats and in humans represent a spectrum of differing biological, anatomical, immunochemical, and experiential factors. While the actor and critic modules may correspond to the dorsal and ventral striatum, respectively, with prediction error related to dopaminergic (Houk, Adams & Barto, 1995) and possibly serotonergic inputs to the striatum (Moutoussis et al., 2008; Seymour et al., 2004), multiple other regions have been implicated in avoidance learning, including the amygdala and prefrontal cortex (Moutoussis et al., 2008). Nevertheless, it is intriguing that these two simple parameters—learning rate and shock cost—suffice to explain data from the lever press avoidance paradigm, and that these parameters do indeed appear to correspond to documented sex differences in rats (Beck et al., 2011) and humans (Sheynin et al., 2014).

Although the current simulations show that the model we selected could account for patterns of behavior, it is of course possible that other computational models may be able to provide as good, or a better fit, to the observed behavior. Therefore, the temporal difference model we selected (based on the actor-critic architecture) may not be in a unique position to explain sex differences in avoidance relative to other reinforcement learning models. Indeed, similar models have been successful in simulating other avoidance learning paradigms (Johnson et al., 2001; Smith et al., 2004; Moutoussis et al., 2008; Maia, 2010). Our choice of modeling framework was instead based on the fact that the selected model captures key elements common to many past and current models of reinforcement learning (Barto, 1995), and as shown by previous work from our laboratory, was capable of making specific predictions about strain differences in escape-avoidance learning observed between SD and WKY rats (Myers et al., 2014). Finally, it could also be extended to account for sex differences in human approach-avoidance learning (Sheynin et al., 2015). We consider it a strength that the same, albeit simple, model can be used to address a wide range of data.

It is also possible that manipulations of alternative parameters in this model could account for the observed sex differences in behavior, which would suggest multiple possible mechanisms that could lead to a given behavioral phenotype. The goal of the current simulations was to identify potential mechanisms that could account for observed sex differences, and generate predictions that could be tested empirically, rather than perform an exhaustive search to determine if other parameter values could replicate the same behavior. Ultimately, additional experimental work is required to test the validity of the model’s predictions, and determine which of these possible mechanisms operate in animals. Also to that end, a final important avenue for future work would be to extend this modeling effort to incorporate biological detail, such as the contribution of sexually dimorphic brain substrates and neurotransmitter systems. Understanding the interplay between the external and internal factors that contribute to sex differences in avoidance learning could shed important light on individual differences in vulnerability to anxiety, which in turn may ultimately lead to improved clinical outcomes.

## Supplemental Information

10.7717/peerj.1081/supp-1 Supplemental Information 1 Values used to construct figures showing intertrial responses Each tab of the file contains values used to construct an individual figure indicated by the name of the tab.

10.7717/peerj.1081/supp-2 Supplemental Information 2 Summary of statistics Complete summary of statistical analyses performed on the avoidance behavior of the model, indexed by experiment and figure.

10.7717/peerj.1081/supp-3 Supplemental Information 3 Values used to construct figures showing weights assigned to each input by the critic or actor module of the model Each tab of the file contains values used to construct a set of figures indicated by the name of the tab.

10.7717/peerj.1081/supp-4 Supplemental Information 4 Values used to construct figures showing proportion avoidance responses Each tab of the file contains values used to construct an individual figure indicated by the name of the tab.

10.7717/peerj.1081/supp-5 Supplemental Information 5 Values used to construct figures showing the latency to avoid Each tab of the file contains values used to construct an individual figure indicated by the name of the tab.
