# Supplementary material for: Using signals associated with safety in avoidance learning: computational model of sex differences
Source: PeerJ. 2015 Jul 14;3:e1081. doi: 10.7717/peerj.1081 (PMC4512772; doi:10.7717/peerj.1081)
Supplement: Supplemental Information 2 — Complete summary of statistical analyses performed on the avoidance behavior of the model, indexed by experiment and figure. [file peerj-03-1081-s002.docx]

**Supplemental Materials**

**1. Experiment 1**

*Table S1.1.* Summary of statistics for Figure 4A.

| **Acquisition*** | **df1** | **df2** | **F** | **p** | **η_p_^2^** |
| --- | --- | --- | --- | --- | --- |
| session | 3.91 | 140.6 | 310.105 | <.001 | .896 |
| sex | 1 | 36 | 161.711 | <.001 | .818 |
| session, sex | 3.91 | 140.6 | 19.589 | <.001 | .352 |
| session, ITI signal | 3.91 | 140.6 | 3.158 | .017 | .081 |
| sex, ITI signal | 1 | 36 | 9.029 | .005 | .201 |
| **Extinction** |  |  |  |  |  |
| session | 11 | 396 | 116.287 | <.001 | .764 |
| sex | 1 | 36 | 213.264 | <.001 | .856 |
| session, sex | 11 | 396 | 4.706 | <.001 | .116 |
| sex, ITI signal | 1 | 36 | 19.01 | <.001 | .346 |
| *Mauchly’s test indicated the assumption of sphericity was violated, χ^2^ (65) = 201, *p*<.001, therefore degrees of freedom were corrected using Greenhouse-Geisser estimates (ε = 0.36). | | | | | |

*Table S1.2.* Summary of statistics for Figure 4B.

| **Acquisition*** | **df1** | **df2** | **F** | **p** | **η_p_^2^** |
| --- | --- | --- | --- | --- | --- |
| session | 4.03 | 145.18 | 317.255 | <.001 | .898 |
| sex | 1 | 36 | 228.857 | <.001 | .864 |
| session, sex | 4.03 | 145.18 | 29.610 | <.001 | .451 |
| **Extinction** |  |  |  |  |  |
| session | 11 | 396 | 157.948 | <.001 | .814 |
| sex | 1 | 36 | 194.589 | <.001 | .844 |
| session, sex | 11 | 396 | 8.286 | <.001 | .187 |
| session, ITI signal | 11 | 396 | 1.865 | .042 | .049 |
| *Mauchly’s test indicated the assumption of sphericity was violated, χ^2^ (65) = 237.8, *p*<.001, therefore degrees of freedom were corrected using Greenhouse-Geisser estimates (ε = 0.37). | | | | | |

*Table S1.3.* Summary of statistics for Figure 4C.

| **Acquisition*** | **df1** | **df2** | **F** | **p** | **η_p_^2^** |
| --- | --- | --- | --- | --- | --- |
| session | 2.6 | 92.1 | 330.703 | <.001 | .902 |
| sex | 1 | 36 | 47.656 | <.001 | .570 |
| session, sex | 2.6 | 92.1 | 15.552 | <.001 | .302 |
| session, sex, ITI signal | 2.6 | 92.1 | 3.448 | .026 | .087 |
| sex, ITI signal | 1 | 36 | 10.902 | .002 | .232 |
| **Extinction**** |  |  |  |  |  |
| session | 8 | 287.4 | 100.7 | <.001 | .737 |
| sex | 1 | 36 | 264.307 | <.001 | .880 |
| session, sex | 8 | 287.4 | 3.039 | .003 | .078 |
| ITI signal | 1 | 36 | 7.981 | .008 | .181 |
| sex, ITI signal | 1 | 36 | 14.712 | <.001 | .290 |
| *Mauchly’s test indicated the assumption of sphericity was violated, χ^2^ (65) = 575.4, *p*<.001, therefore degrees of freedom were corrected using Greenhouse-Geisser estimates (ε = 0.23).  **Mauchly’s test indicated the assumption of sphericity was violated, χ^2^ (65) = 99.3, *p*=.005, therefore degrees of freedom were corrected using Greenhouse-Geisser estimates (ε = 0.73). | | | | | |

*Table S1.4.* Summary of statistics for Figure 4D.

| **Acquisition*** | **df1** | **df2** | **F** | **p** | **η_p_^2^** |
| --- | --- | --- | --- | --- | --- |
| session | 3 | 108.3 | 266.56 | <.001 | .881 |
| sex | 1 | 36 | 40.208 | <.001 | .528 |
| session, sex | 3 | 108.3 | 19.799 | <.001 | .355 |
| **Extinction**** |  |  |  |  |  |
| session | 7.3 | 265.7 | 111.83 | <.001 | .756 |
| sex | 1 | 36 | 67.35 | <.001 | .652 |
| session, sex | 7.3 | 265.7 | 5.382 | <.001 | .130 |
| *Mauchly’s test indicated the assumption of sphericity was violated, χ^2^ (65) = 620.4, *p*<.001, therefore degrees of freedom were corrected using Greenhouse-Geisser estimates (ε = 0.27).  **Mauchly’s test indicated the assumption of sphericity was violated, χ^2^ (65) = 120.8, *p*<.001, therefore degrees of freedom were corrected using Greenhouse-Geisser estimates (ε = 0.67). | | | | | |

**2. Experiment 2**

*Table S2.1.* Summary of statistics for Figure 7A.

| **Acquisition*** | **df1** | **df2** | **F** | **p** | **η_p_^2^** |
| --- | --- | --- | --- | --- | --- |
| session | 4.22 | 152.01 | 347.208 | <.001 | .906 |
| shock cost | 1 | 36 | 140.556 | <.001 | .796 |
| ITI signal | 1 | 36 | 10.177 | .003 | .220 |
| session, shock cost | 4.22 | 152.01 | 28.434 | <.001 | .441 |
| session, ITI signal | 4.22 | 152.01 | 2.573 | .037 | .067 |
| **Extinction** |  |  |  |  |  |
| session | 11 | 396 | 131.639 | <.001 | .785 |
| shock cost | 1 | 36 | 22.463 | <.001 | .384 |
| ITI signal | 1 | 36 | 25.481 | <.001 | .414 |
| *Mauchly’s test indicated the assumption of sphericity was violated, χ^2^ (65) = 219, *p*<.001, therefore degrees of freedom were corrected using Greenhouse-Geisser estimates (ε = 0.38). | | | | | |

*Table S2.2.* Summary of statistics for Figure 7B.

| **Acquisition*** | **df1** | **df2** | **F** | **p** | **η_p_^2^** |
| --- | --- | --- | --- | --- | --- |
| session | 4.94 | 177.67 | 279.428 | <.001 | .886 |
| shock cost | 1 | 36 | 91.3 | <.001 | .717 |
| ITI signal | 1 | 36 | 12.084 | <.001 | .251 |
| session, shock cost | 4.94 | 177.67 | 22.434 | <.001 | .384 |
| session, ITI signal | 4.94 | 177.67 | 3.747 | .003 | .094 |
| session, shock cost, ITI signal | 4.94 | 177.67 | 3.550 | .005 | .090 |
| **Extinction** |  |  |  |  |  |
| session | 11 | 396 | 136.676 | <.001 | .792 |
| shock cost | 1 | 36 | 46.139 | <.001 | .562 |
| session, shock cost | 11 | 396 | 1.962 | .031 | .052 |
| *Mauchly’s test indicated the assumption of sphericity was violated, χ^2^ (65) = 177.8, *p*<.001, therefore degrees of freedom were corrected using Greenhouse-Geisser estimates (ε = 0.45). | | | | | |

*Table S2.3.* Summary of statistics for Figure 7C.

| **Acquisition*** | **df1** | **df2** | **F** | **p** | **η_p_^2^** |
| --- | --- | --- | --- | --- | --- |
| session | 5.56 | 200.12 | 328.897 | <.001 | .901 |
| ITI signal | 1 | 36 | 8.425 | .006 | .190 |
| session, α | 5.56 | 200.12 | 2.207 | .048 | .058 |
| session, ITI signal | 5.56 | 200.12 | 2.426 | .031 | .063 |
| **Extinction** |  |  |  |  |  |
| session | 11 | 396 | 118.899 | <.001 | .768 |
| α | 1 | 36 | 75.968 | <.001 | .678 |
| ITI signal | 1 | 36 | 5.796 | .021 | .139 |
| session, α | 11 | 396 | 6.358 | <.001 | .150 |
| α, ITI signal | 1 | 36 | 8.382 | .006 | .189 |
| *Mauchly’s test indicated the assumption of sphericity was violated, χ^2^ (65) = 154.5, *p*<.001, therefore degrees of freedom were corrected using Greenhouse-Geisser estimates (ε = 0.51). | | | | | |

*Table S2.4.* Summary of statistics for Figure 7D.

| **Acquisition*** | **df1** | **df2** | **F** | **p** | **η_p_^2^** |
| --- | --- | --- | --- | --- | --- |
| session | 2.53 | 91.16 | 297.396 | <.001 | .892 |
| α | 1 | 36 | 14.711 | <.001 | .290 |
| **Extinction** |  |  |  |  |  |
| session | 11 | 396 | 180.222 | <.001 | .834 |
| α | 1 | 36 | 68.085 | <.001 | .654 |
| ITI signal | 1 | 36 | 17.843 | <.001 | .331 |
| session, α | 11 | 396 | 3.965 | <.001 | .099 |
| session, ITI signal | 11 | 396 | 1.866 | .042 | .049 |
| session, α, ITI signal | 11 | 396 | 2.322 | .009 | .061 |
| *Mauchly’s test indicated the assumption of sphericity was violated, χ^2^ (65) = 301.3, *p*<.001, therefore degrees of freedom were corrected using Greenhouse-Geisser estimates (ε = .23). | | | | | |

**3. Experiment 3**

*Table S3.1.* Summary of statistics for Figure 8A.

| **Acquisition*** | **df1** | **df2** | **F** | **p** | **η_p_^2^** |
| --- | --- | --- | --- | --- | --- |
| session | 3.98 | 143.38 | 301.67 | <.001 | .893 |
| sex | 1 | 36 | 185.07 | <.001 | .837 |
| ITI signal | 1 | 36 | 16.757 | <.001 | .318 |
| session, sex | 3.98 | 143.38 | 18.955 | <.001 | .345 |
| session, ITI signal | 3.98 | 143.38 | 2.525 | .044 | .066 |
| **Extinction** |  |  |  |  |  |
| session | 11 | 396 | 56.898 | <.001 | .612 |
| sex | 1 | 36 | 196.248 | <.001 | .845 |
| ITI signal | 1 | 36 | 7.431 | .010 | .171 |
| session, sex | 11 | 396 | 5.575 | <.001 | .134 |
| sex, ITI signal | 1 | 36 | 11.536 | .002 | .243 |
| *Mauchly’s test indicated the assumption of sphericity was violated, χ^2^ (65) = 239.5, *p*<.001, therefore degrees of freedom were corrected using Greenhouse-Geisser estimates (ε = 0.36). | | | | | |

*Table S3.2.* Summary of statistics for Figure 8B.

| **Acquisition*** | **df1** | **df2** | **F** | **p** | **η_p_^2^** |
| --- | --- | --- | --- | --- | --- |
| session | 3 | 107 | 331.1 | <.001 | .902 |
| sex | 1 | 36 | 49.59 | <.001 | .579 |
| ITI signal | 1 | 36 | 8.520 | .006 | .191 |
| session, sex | 3 | 107 | 15.012 | <.001 | .294 |
| session, ITI signal | 3 | 107 | 4.47 | .006 | .110 |
| **Extinction** |  |  |  |  |  |
| session | 11 | 396 | 65.979 | <.001 | .647 |
| sex | 1 | 36 | 256.26 | <.001 | .877 |
| ITI signal | 1 | 36 | 6.24 | .017 | .148 |
| session, sex | 11 | 396 | 3.437 | <.001 | .087 |
| sex, ITI signal | 1 | 36 | 9.19 | .004 | .203 |
| *Mauchly’s test indicated the assumption of sphericity was violated, χ^2^ (65) = 564.4, *p*<.001, therefore degrees of freedom were corrected using Greenhouse-Geisser estimates (ε = 0.27). | | | | | |

**4. Experiment 4**

*Table S4.1.* Summary of statistics for Figure 9A.

| **Acquisition*** | **df1** | **df2** | **F** | **p** | **η_p_^2^** |
| --- | --- | --- | --- | --- | --- |
| session | 4.39 | 158.15 | 248.148 | <.001 | .873 |
| sex | 1 | 36 | 123.247 | <.001 | .774 |
| ITI signal | 1 | 36 | 12.108 | .001 | .252 |
| session, sex | 4.39 | 158.15 | 15.583 | <.001 | .302 |
| **Extinction**** |  |  |  |  |  |
| session | 6.62 | 238.26 | 93.301 | <.001 | .722 |
| sex | 1 | 36 | 512.523 | <.001 | .934 |
| ITI signal | 1 | 36 | 11.705 | .002 | .245 |
| session, sex | 6.62 | 238.26 | 12.523 | <.001 | .258 |
| sex, ITI signal | 1 | 36 | 12.723 | .001 | .261 |
| *Mauchly’s test indicated the assumption of sphericity was violated, χ^2^ (65) = 245.3, *p*<.001, therefore degrees of freedom were corrected using Greenhouse-Geisser estimates (ε = 0.40).  **Mauchly’s test indicated the assumption of sphericity was violated, χ^2^ (65) = 151.2, *p*<.001, therefore degrees of freedom were corrected using Greenhouse-Geisser estimates (ε = 0.60).  *Table S4.2.* Summary of statistics for Figure 9B.   \| **Acquisition*** \| **df1** \| **df2** \| **F** \| **p** \| **η_p_^2^** \| \| --- \| --- \| --- \| --- \| --- \| --- \| \| session \| 3.4 \| 123.1 \| 206.2 \| <.001 \| .851 \| \| sex \| 1 \| 36 \| 67.7 \| <.001 \| .653 \| \| ITI signal \| 1 \| 36 \| 7.8 \| .008 \| .178 \| \| session, sex \| 3.4 \| 123.1 \| 11.5 \| <.001 \| .242 \| \| **Extinction**** \|  \|  \|  \|  \|  \| \| session \| 6.4 \| 230.4 \| 67.05 \| <.001 \| .651 \| \| sex \| 1 \| 36 \| 404.26 \| <.001 \| .918 \| \| ITI signal \| 1 \| 36 \| 8.74 \| .005 \| .195 \| \| session, sex \| 6.4 \| 230.4 \| 11.672 \| <.001 \| .245 \| \| sex, ITI signal \| 1 \| 36 \| 7.4 \| .01 \| .171 \| \| *Mauchly’s test indicated the assumption of sphericity was violated, χ^2^ (65) = 497.5, *p*<.001, therefore degrees of freedom were corrected using Greenhouse-Geisser estimates (ε = 0.31).  **Mauchly’s test indicated the assumption of sphericity was violated, χ^2^ (65) = 187.2, *p*<.001, therefore degrees of freedom were corrected using Greenhouse-Geisser estimates (ε = 0.58). \| \| \| \| \| \| | | | | | |

*Table S4.3.* Summary of statistics for Figure 10A (ITI duration = 30 seconds).

| **Acquisition** | **df1** | **df2** | **F** | **p** | **η_p_^2^** |
| --- | --- | --- | --- | --- | --- |
| segment | 2 | 72 | 44.404 | <.001 | .552 |
| sex | 1 | 36 | 465.231 | <.001 | .928 |
| ITI signal | 1 | 36 | 15.634 | <.001 | .303 |

*Table S4.4.* Summary of statistics for Figure 10B (ITI duration = 180 seconds).

| **Acquisition** | **df1** | **df2** | **F** | **p** | **η_p_^2^** |
| --- | --- | --- | --- | --- | --- |
| segment | 2 | 72 | 422.958 | <.001 | .922 |
| sex | 1 | 36 | 213.928 | <.001 | .856 |
| sex, ITI signal | 1 | 36 | 12.294 | .001 | .255 |
| segment, sex | 2 | 72 | 8.319 | .001 | .188 |

**5. Experiment 5**

*Table S5.1.* Summary of statistics for the overall analysis of proportion avoidance.

| **Acquisition*** | **df1** | **df2** | **F** | **p** | **η_p_^2^** |
| --- | --- | --- | --- | --- | --- |
| session | 5.13 | 369.66 | 659.120 | <.001 | .902 |
| sex | 1 | 72 | 307.482 | <.001 | .810 |
| ITI duration | 1 | 72 | 129.363 | <.001 | .642 |
| session, sex | 5.13 | 369.66 | 50.795 | <.001 | .414 |
| session, ITI duration | 5.13 | 369.66 | 22.299 | <.001 | .236 |
| session, ITI signal | 5.13 | 369.66 | 3.457 | .004 | .046 |
| sex, ITI duration | 1 | 72 | 15.907 | <.001 | .181 |
| ITI duration, ITI signal | 1 | 72 | 36.287 | <.001 | .335 |
| session, sex, ITI duration | 5.13 | 369.66 | 19.128 | <.001 | .210 |
| session, sex, ITI signal | 5.13 | 369.66 | 3.253 | .006 | .043 |
| session, ITI duration, ITI signal | 5.13 | 369.66 | 5.710 | <.001 | .073 |
| sex, ITI duration, ITI signal | 1 | 72 | 16.910 | <.001 | .190 |
| session, sex, ITI duration, ITI signal | 5.13 | 369.66 | 5.518 | <.001 | .071 |
| **Extinction**** |  |  |  |  |  |
| session | 8.1 | 582.7 | 262.592 | <.001 | .785 |
| sex | 1 | 72 | 936.534 | <.001 | .929 |
| ITI duration | 1 | 72 | 427.055 | <.001 | .856 |
| session, sex | 8.1 | 582.7 | 12.979 | <.001 | .153 |
| session, ITI duration | 8.1 | 582.7 | 12.495 | <.001 | .148 |
| ITI duration, ITI signal | 1 | 72 | 15.613 | <.001 | .178 |
| session, sex, ITI duration | 8.1 | 582.7 | 17.822 | <.001 | .198 |
| sex, ITI duration, ITI signal | 1 | 72 | 5.810 | .018 | .075 |
| session, ITI duration, ITI signal | 8.1 | 582.7 | 2.442 | .013 | .033 |
| session, sex, ITI duration, ITI signal | 8.1 | 582.7 | 2.998 | .003 | .040 |
| *Mauchly’s test indicated the assumption of sphericity was violated, χ^2^ (65) = 384.2, *p*<.001, therefore degrees of freedom were corrected using Greenhouse-Geisser estimates (ε = 0.47).  **Mauchly’s test indicated the assumption of sphericity was violated, χ^2^ (65) = 141.6, *p*<.001, therefore degrees of freedom were corrected using Greenhouse-Geisser estimates (ε = 0.74). | | | | | |

*Table S5.2.* Summary of statistics for Figure 11A (ITI duration = 180 seconds).

| **Acquisition*** | **df1** | **df2** | **F** | **p** | **η_p_^2^** |
| --- | --- | --- | --- | --- | --- |
| session | 3.58 | 128.8 | 332.079 | <.001 | .902 |
| sex | 1 | 36 | 165.547 | <.001 | .821 |
| ITI signal | 1 | 36 | 13.026 | .001 | .266 |
| session, sex | 3.58 | 128.8 | 36.514 | <.001 | .504 |
| session, ITI signal | 3.58 | 128.8 | 4.812 | .002 | .118 |
| sex, ITI signal | 1 | 36 | 7.768 | .008 | .266 |
| session, sex, ITI signal | 3.58 | 128.8 | 4.817 | .002 | .118 |
| **Extinction** |  |  |  |  |  |
| session | 11 | 396 | 197.710 | <.001 | .846 |
| sex | 1 | 36 | 505.715 | <.001 | .934 |
| ITI signal | 1 | 36 | 12.926 | .001 | .264 |
| session, sex | 11 | 396 | 9.442 | <.001 | .208 |
| session, sex, ITI signal | 11 | 396 | 1.885 | .040 | .050 |
| *Mauchly’s test indicated the assumption of sphericity was violated, χ^2^ (65) = 289.6, *p*<.001, therefore degrees of freedom were corrected using Greenhouse-Geisser estimates (ε = 0.33). | | | | | |

*Table S5.3.* Summary of statistics for Figure 11B (ITI duration = 30 seconds).

| **Acquisition*** | **df1** | **df2** | **F** | **p** | **η_p_^2^** |
| --- | --- | --- | --- | --- | --- |
| session | 4.19 | 150.69 | 353.439 | <.001 | .908 |
| sex | 1 | 36 | 152.725 | <.001 | .809 |
| ITI signal | 1 | 36 | 30.061 | <.001 | .455 |
| session, sex | 4.19 | 150.69 | 32.672 | <.001 | .476 |
| session, ITI signal | 4.19 | 150.69 | 4.246 | .002 | .106 |
| sex, ITI signal | 1 | 36 | 10.559 | .003 | .227 |
| session, sex, ITI signal | 4.19 | 150.69 | 3.748 | .005 | .094 |
| **Extinction**** |  |  |  |  |  |
| session | 6.22 | 223.77 | 81.536 | <.001 | .694 |
| sex | 1 | 36 | 432.972 | <.001 | .923 |
| session, sex | 6.22 | 223.77 | 20.948 | <.001 | .368 |
| session, ITI signal | 6.22 | 223.77 | 2.642 | .016 | .068 |
| *Mauchly’s test indicated the assumption of sphericity was violated, χ^2^ (65) = 283.8, *p*<.001, therefore degrees of freedom were corrected using Greenhouse-Geisser estimates (ε = 0.38).  **Mauchly’s test indicated the assumption of sphericity was violated, χ^2^ (65) = 178.5, *p*<.001, therefore degrees of freedom were corrected using Greenhouse-Geisser estimates (ε = 0.57). | | | | | |

*Table S5.4.* Summary of statistics for the overall analysis of latency to press the lever.

| **Acquisition*** | **df1** | **df2** | **F** | **p** | **η_p_^2^** |
| --- | --- | --- | --- | --- | --- |
| session | 4.4 | 319.8 | 543.99 | <.001 | .883 |
| sex | 1 | 72 | 126.77 | <.001 | .638 |
| ITI duration | 1 | 72 | 130.46 | <.001 | .644 |
| session, sex | 4.4 | 319.8 | 40 | <.001 | .357 |
| session, ITI duration | 4.4 | 319.8 | 50.39 | <.001 | .412 |
| session, ITI signal | 4.4 | 319.8 | 2.58 | .032 | .035 |
| sex, ITI duration | 1 | 72 | 13.71 | <.001 | .160 |
| ITI duration, ITI signal | 1 | 72 | 19.03 | <.001 | .209 |
| session, sex, ITI duration | 4.4 | 319.8 | 10.34 | <.001 | .126 |
| session, ITI duration, ITI signal | 4.4 | 319.8 | 6.05 | <.001 | .078 |
| sex, ITI duration, ITI signal | 1 | 72 | 9.61 | .003 | .118 |
| session, sex, ITI duration, ITI signal | 4.4 | 319.8 | 4.995 | <.001 | .065 |
| **Extinction**** |  |  |  |  |  |
| session | 6.6 | 474.5 | 232.89 | <.001 | .764 |
| sex | 1 | 72 | 786.6 | <.001 | .916 |
| ITI duration | 1 | 72 | 324.1 | <.001 | .818 |
| session, sex | 6.6 | 474.5 | 30.92 | <.001 | .300 |
| session, ITI duration | 6.6 | 474.5 | 28.81 | <.001 | .286 |
| ITI duration, ITI signal | 1 | 72 | 13.52 | <.001 | .158 |
| session, sex, ITI duration | 6.6 | 474.5 | 3.57 | .001 | .047 |
| sex, ITI duration | 1 | 72 | 16.35 | <.001 | .185 |
| session, ITI duration, ITI signal | 6.6 | 474.5 | 2.31 | .028 | .031 |
| session, sex, ITI duration, ITI signal | 6.6 | 474.5 | 3.4 | .002 | .045 |
| *Mauchly’s test indicated the assumption of sphericity was violated, χ^2^ (65) = 890.9, *p*<.001, therefore degrees of freedom were corrected using Greenhouse-Geisser estimates (ε = 0.4).  **Mauchly’s test indicated the assumption of sphericity was violated, χ^2^ (65) = 333, *p*<.001, therefore degrees of freedom were corrected using Greenhouse-Geisser estimates (ε = 0.6). | | | | | |

*Table S5.5.* Summary of statistics for Figure 11C (ITI duration = 180 seconds).

| **Acquisition*** | **df1** | **df2** | **F** | **p** | **η_p_^2^** |
| --- | --- | --- | --- | --- | --- |
| session | 3.6 | 128.6 | 311.42 | <.001 | .896 |
| sex | 1 | 36 | 66.89 | <.001 | .650 |
| ITI signal | 1 | 36 | 7.87 | .008 | .179 |
| session, sex | 3.6 | 128.6 | 27.76 | <.001 | .435 |
| session, ITI signal | 3.6 | 128.6 | 4.55 | .003 | .112 |
| sex, ITI signal | 1 | 36 | 5.2 | .029 | .126 |
| session, sex, ITI signal | 3.6 | 128.6 | 3.12 | .021 | .080 |
| **Extinction**** |  |  |  |  |  |
| session | 6.4 | 229.6 | 190.67 | <.001 | .841 |
| sex | 1 | 36 | 414.48 | <.001 | .920 |
| ITI signal | 1 | 36 | 12.43 | .001 | .257 |
| session, sex | 6.4 | 229.6 | 14.58 | <.001 | .288 |
| session, sex, ITI signal | 6.4 | 229.6 | 2.9 | .008 | .075 |
| *Mauchly’s test indicated the assumption of sphericity was violated, χ^2^ (65) = 572.1, *p*<.001, therefore degrees of freedom were corrected using Greenhouse-Geisser estimates (ε = 0.33).  **Mauchly’s test indicated the assumption of sphericity was violated, χ^2^ (65) = 213.3, *p*<.001, therefore degrees of freedom were corrected using Greenhouse-Geisser estimates (ε = 0.58). | | | | | |

*Table S5.6.* Summary of statistics for Figure 11D (ITI duration = 30 seconds).

| **Acquisition*** | **df1** | **df2** | **F** | **p** | **η_p_^2^** |
| --- | --- | --- | --- | --- | --- |
| session | 3.9 | 141.8 | 262.02 | <.001 | .879 |
| sex | 1 | 36 | 87.4 | <.001 | .708 |
| ITI signal | 1 | 36 | 19.74 | <.001 | .354 |
| session, sex | 3.9 | 141.8 | 18.78 | <.001 | .343 |
| session, ITI signal | 3.9 | 141.8 | 3.76 | .006 | .095 |
| sex, ITI signal | 1 | 36 | 6.3 | .017 | .149 |
| session, sex, ITI signal | 3.9 | 141.8 | 3.2 | .015 | .082 |
| **Extinction**** |  |  |  |  |  |
| session | 5.3 | 190.3 | 56.21 | <.001 | .610 |
| sex | 1 | 36 | 380.16 | <.001 | .913 |
| session, sex | 5.3 | 190.3 | 20.58 | <.001 | .364 |
| *Mauchly’s test indicated the assumption of sphericity was violated, χ^2^ (65) = 533.2, *p*<.001, therefore degrees of freedom were corrected using Greenhouse-Geisser estimates (ε = 0.36).  **Mauchly’s test indicated the assumption of sphericity was violated, χ^2^ (65) = 274.5, *p*<.001, therefore degrees of freedom were corrected using Greenhouse-Geisser estimates (ε = 0.48). | | | | | |

**6. Experiment 6**

*Table S6.1.* Summary of statistics for the overall analysis of proportion avoidance.

| **Acquisition*** | **df1** | **df2** | **F** | **p** | **η_p_^2^** |
| --- | --- | --- | --- | --- | --- |
| session | 4.4 | 313.8 | 564.335 | <.001 | .887 |
| sex | 1 | 72 | 314.747 | <.001 | .814 |
| session, sex | 4.4 | 313.8 | 41.342 | <.001 | .365 |
| sex, ITI timing | 1 | 72 | 4.676 | .034 | .061 |
| **Extinction** |  |  |  |  |  |
| session | 11 | 792 | 237.104 | <.001 | .767 |
| sex | 1 | 72 | 374.643 | <.001 | .839 |
| session, sex | 11 | 792 | 11.624 | <.001 | .139 |
| sex, ITI signal, ITI timing | 1 | 72 | 8.056 | .006 | .101 |
| *Mauchly’s test indicated the assumption of sphericity was violated, χ^2^ (65) = 355.2, *p*<.001, therefore degrees of freedom were corrected using Greenhouse-Geisser estimates (ε = 0.40). | | | | | |

*Table S6.2.* Summary of statistics for Figure 14A (ITI signal in the first 30 seconds of the ITI).

| **Acquisition*** | **df1** | **df2** | **F** | **p** | **η_p_^2^** |
| --- | --- | --- | --- | --- | --- |
| session | 3.8 | 135.1 | 266.755 | <.001 | .881 |
| sex | 1 | 36 | 120.151 | <.001 | .769 |
| session, sex | 3.8 | 135.1 | 16.136 | <.001 | .309 |
| **Extinction** |  |  |  |  |  |
| session | 11 | 396 | 126.192 | <.001 | .778 |
| sex | 1 | 36 | 203.667 | <.001 | .850 |
| session, sex | 11 | 396 | 5.603 | <.001 | .135 |
| sex, ITI signal | 1 | 36 | 12.802 | <.001 | .262 |
| *Mauchly’s test indicated the assumption of sphericity was violated, χ^2^ (65) = 258.1, *p*<.001, therefore degrees of freedom were corrected using Greenhouse-Geisser estimates (ε = 0.34). | | | | | |

*Table S6.3.* Summary of statistics for Figure 14B (ITI signal in the last 30 seconds of the ITI).

| **Acquisition*** | **df1** | **df2** | **F** | **p** | **η_p_^2^** |
| --- | --- | --- | --- | --- | --- |
| session | 4.55 | 163.76 | 300.861 | <.001 | .893 |
| sex | 1 | 36 | 200.068 | <.001 | .848 |
| session, sex | 4.55 | 163.76 | 26.714 | <.001 | .426 |
| **Extinction** |  |  |  |  |  |
| session | 11 | 396 | 112.447 | <.001 | .757 |
| sex | 1 | 36 | 175.431 | <.001 | .830 |
| session, sex | 11 | 396 | 6.949 | <.001 | .162 |
| *Mauchly’s test indicated the assumption of sphericity was violated, χ^2^ (65) = 185.6, *p*<.001, therefore degrees of freedom were corrected using Greenhouse-Geisser estimates (ε = 0.41). | | | | | |

*Table S6.4.* Summary of statistics for the overall analysis of latency to press the lever.

| **Acquisition*** | **df1** | **df2** | **F** | **p** | **η_p_^2^** |
| --- | --- | --- | --- | --- | --- |
| session | 3.1 | 220.9 | 523.33 | <.001 | .879 |
| sex | 1 | 72 | 91.55 | <.001 | .560 |
| session, sex | 3.1 | 220.9 | 29.56 | <.001 | .291 |
| **Extinction**** |  |  |  |  |  |
| session | 8.3 | 595.4 | 215.36 | <.001 | .749 |
| sex | 1 | 72 | 456.89 | <.001 | .864 |
| session, sex | 8.3 | 595.4 | 9.5 | <.001 | .117 |
| sex, ITI signal, ITI timing | 1 | 72 | 6.28 | .014 | .080 |
| *Mauchly’s test indicated the assumption of sphericity was violated, χ^2^ (65) = 1033.4, *p*<.001, therefore degrees of freedom were corrected using Greenhouse-Geisser estimates (ε = 0.28).  **Mauchly’s test indicated the assumption of sphericity was violated, χ^2^ (65) = 184.48, *p*<.001, therefore degrees of freedom were corrected using Greenhouse-Geisser estimates (ε = 0.75). | | | | | |

*Table S6.5.* Summary of statistics for Figure 14C (ITI signal in the first 30 seconds of the ITI).

| **Extinction** | **df1** | **df2** | **F** | **p** | **η_p_^2^** |
| --- | --- | --- | --- | --- | --- |
| session | 7.3 | 261.9 | 109.96 | <.001 | .753 |
| sex | 1 | 36 | 222.01 | <.001 | .860 |
| session, sex | 7.3 | 261.9 | 4.49 | <.001 | .111 |
| sex, ITI signal | 1 | 36 | 8.82 | .005 | .197 |
| *Mauchly’s test indicated the assumption of sphericity was violated, χ^2^ (65) = 122.91, *p*<.001, therefore degrees of freedom were corrected using Greenhouse-Geisser estimates (ε = 0.66). | | | | | |

*Table S6.6.* Summary of statistics for Figure 14D (ITI signal in the last 30 seconds of the ITI).

| **Extinction** | **df1** | **df2** | **F** | **p** | **η_p_^2^** |
| --- | --- | --- | --- | --- | --- |
| session | 7.1 | 257.4 | 106.37 | <.001 | .747 |
| sex | 1 | 36 | 234.97 | <.001 | .867 |
| session, sex | 7.1 | 257.4 | 5.75 | <.001 | .138 |
| *Mauchly’s test indicated the assumption of sphericity was violated, χ^2^ (65) = 120.13, *p*<.001, therefore degrees of freedom were corrected using Greenhouse-Geisser estimates (ε = 0.65). | | | | | |
